# Supplementary material for: Cryo-EM structures of NHEJ assemblies with nucleosomes
Source: Nat Commun. 2025 Dec 24;17:648. doi: 10.1038/s41467-025-67376-2 (PMC12816671; doi:10.1038/s41467-025-67376-2)
Supplement: Supplementary file 1 — Supplementary Information [file 41467_2025_67376_MOESM1_ESM.pdf]

Supplementary Information

Cryo-EM structures of NHEJ assemblies with nucleosomes

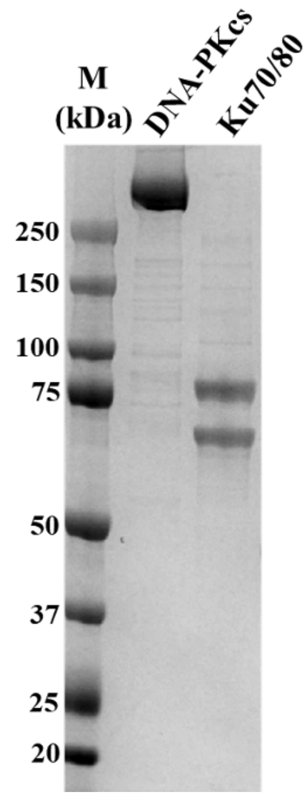

Supplementary Figure 1: DNA-PKcs and Ku70/80 protein purity SDS-PAGE gel.

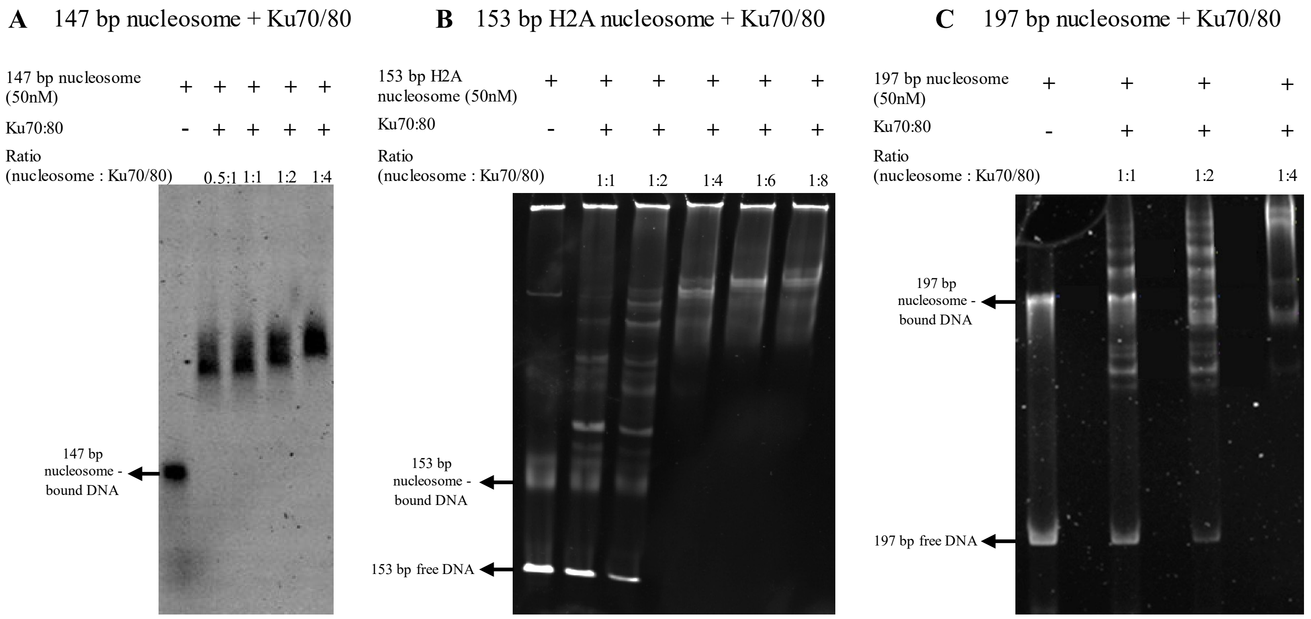

Supplementary Figure 2: Ku70/80 and nucleosome EMSAs. A) 147 bp nucleosome and Ku70/80 EMSA, **B)** 153 bp H2A nucleosome and Ku70/80 EMSA and **C)** 197 bp nucleosome and Ku70/80 EMSA**.** In each EMSA, the nucleosome concentration was kept constant (50nM), and Ku70/80 was titrated at varying ratios displayed. Bands corresponding to free and nucleosome-bound DNA are labelled.

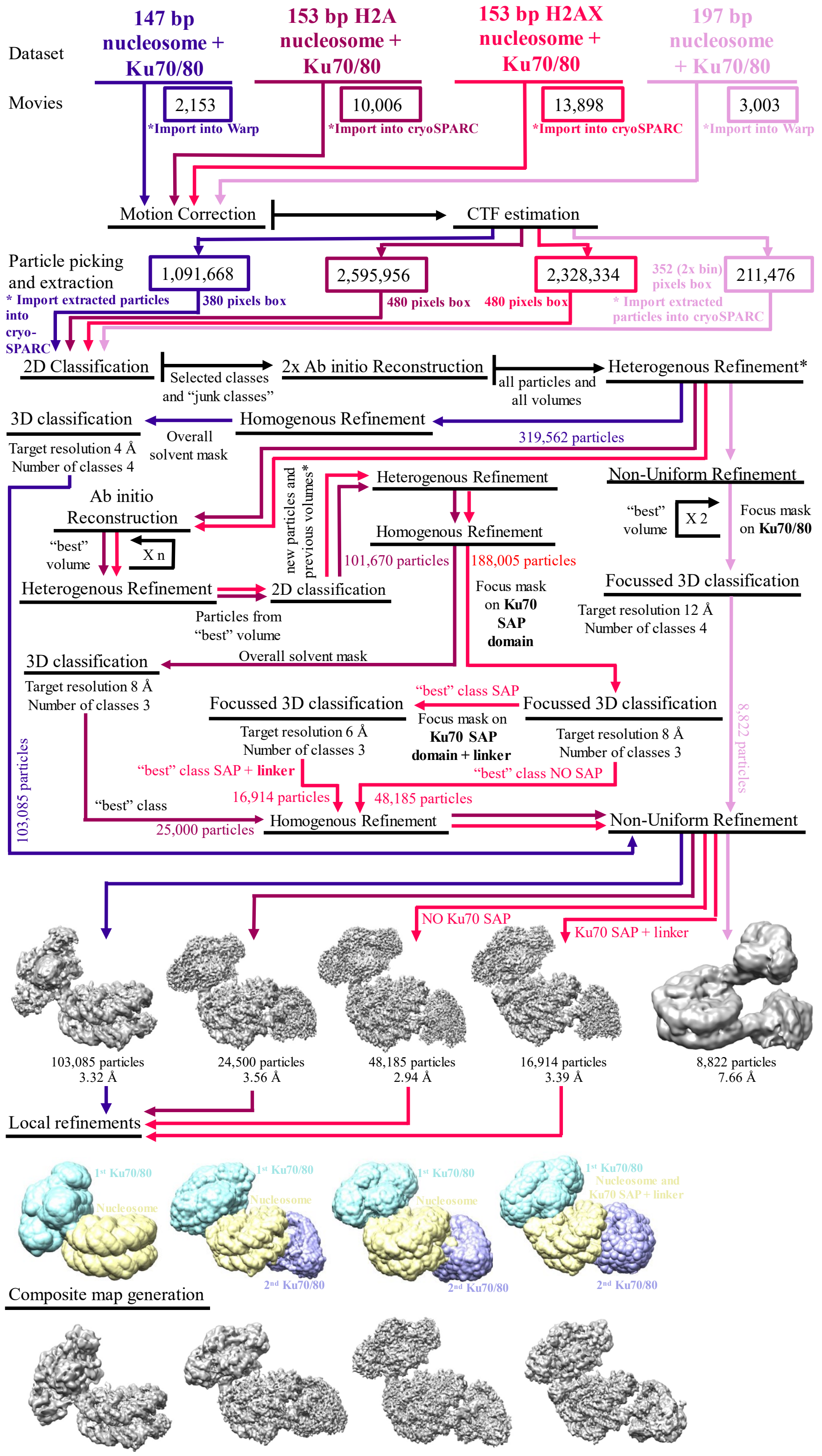

Supplementary Figure 3: Single particle cryo-EM data processing workflows for the Ku70/80 bound to nucleosome maps. For the 147 bp and 197 bp nucleosome and Ku70/80 datasets, the motion correction, CTF estimation and particle picking were completed in Warp; following this, the particle sets were imported into CryoSPARC. For the 153 bp H2A and H2AX nucleosome and Ku70/80 datasets, the entire processing pipeline was completed in CryoSPARC. For all datasets, the picked particles were subject to 2D classification, and following this, the "good" and "junk" 2D classes were used to generate 3D models via *ab initio reconstruction.* A heterogeneous refinement was then used with all particles and volumes. After this stage, varying refinements and classifications were completed to produce a final non-uniform map. The number of particles and resolution for an FSC of 0.143 are shown for each final map. In most cases (except for the 197 bp data), regions were locally refined and used to produce composite maps.

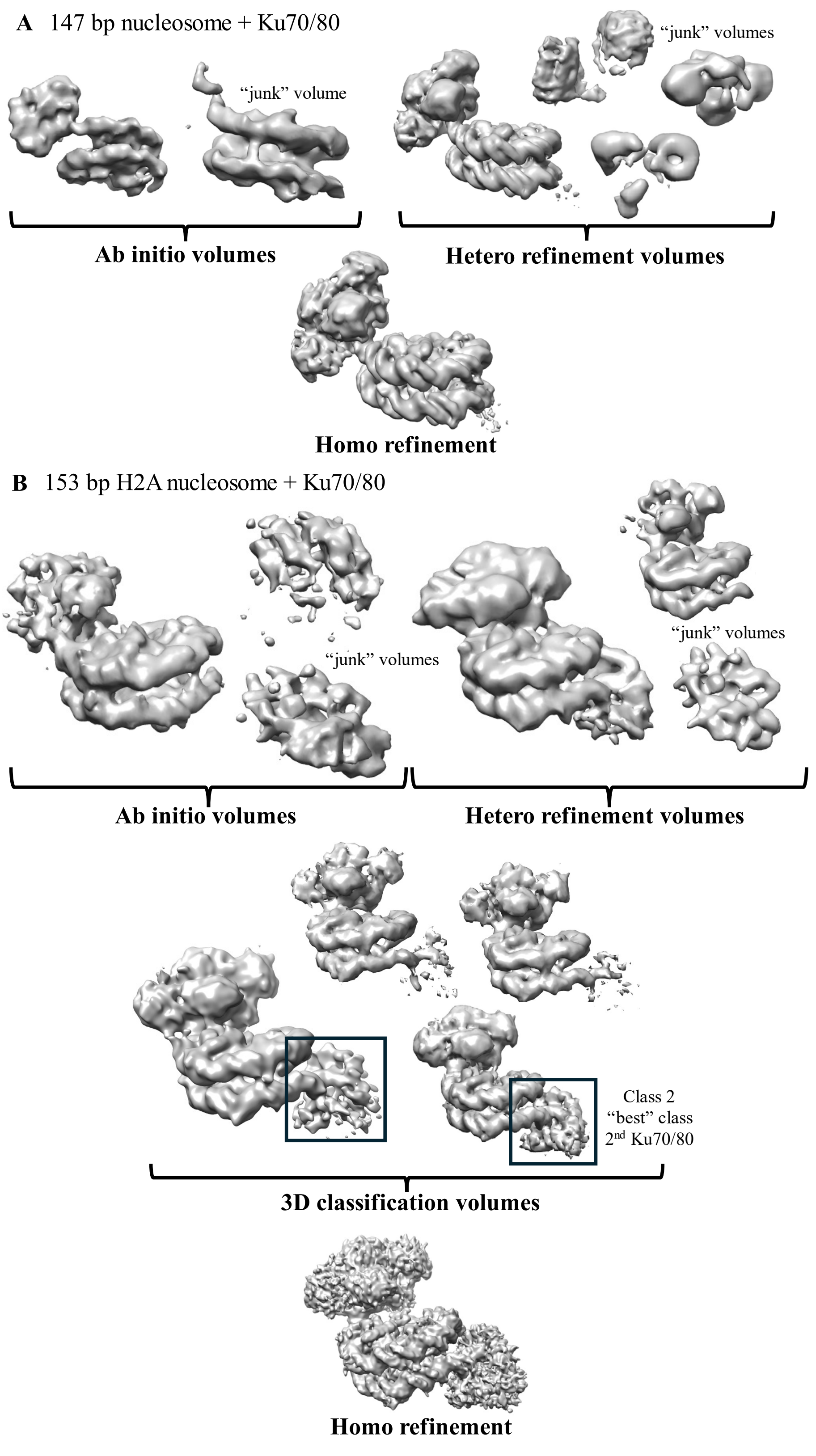

Supplementary Figure 4: Intermediate maps from data processing of the 147 bp and 153 bp H2A nucleosome + Ku70/80 datasets. Volumes from *ab initio reconstruction*, heterogeneous refinements, 3D classification and homogenous refinement are shown, including "junk" volumes.

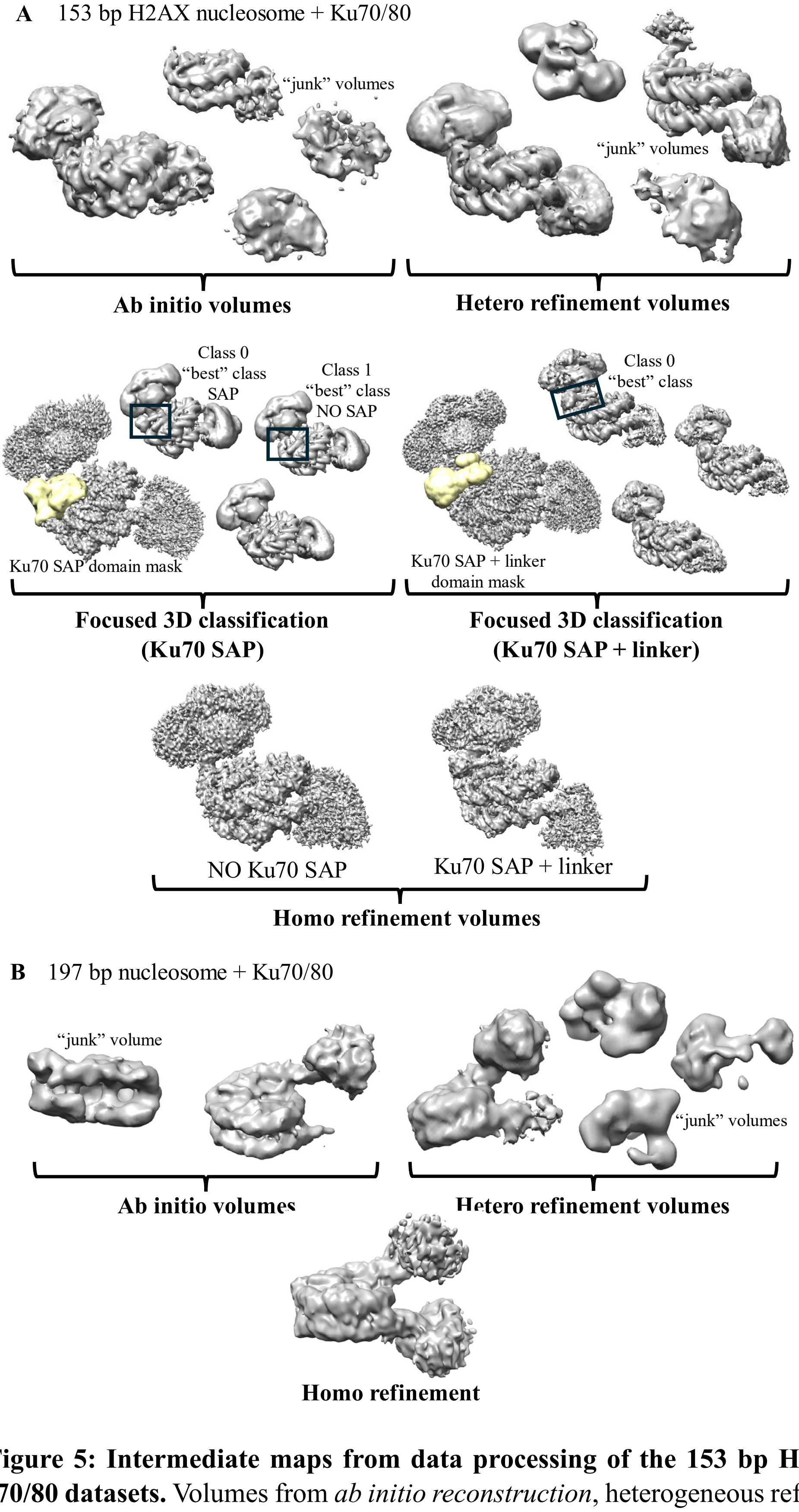

Supplementary Figure 5: Intermediate maps from data processing of the 153 bp H2AX and 197 bp nucleosome + Ku70/80 datasets. Volumes from *ab initio reconstruction*, heterogeneous refinements, focused 3D classification, and homogenous refinement are shown, including "junk" volumes.

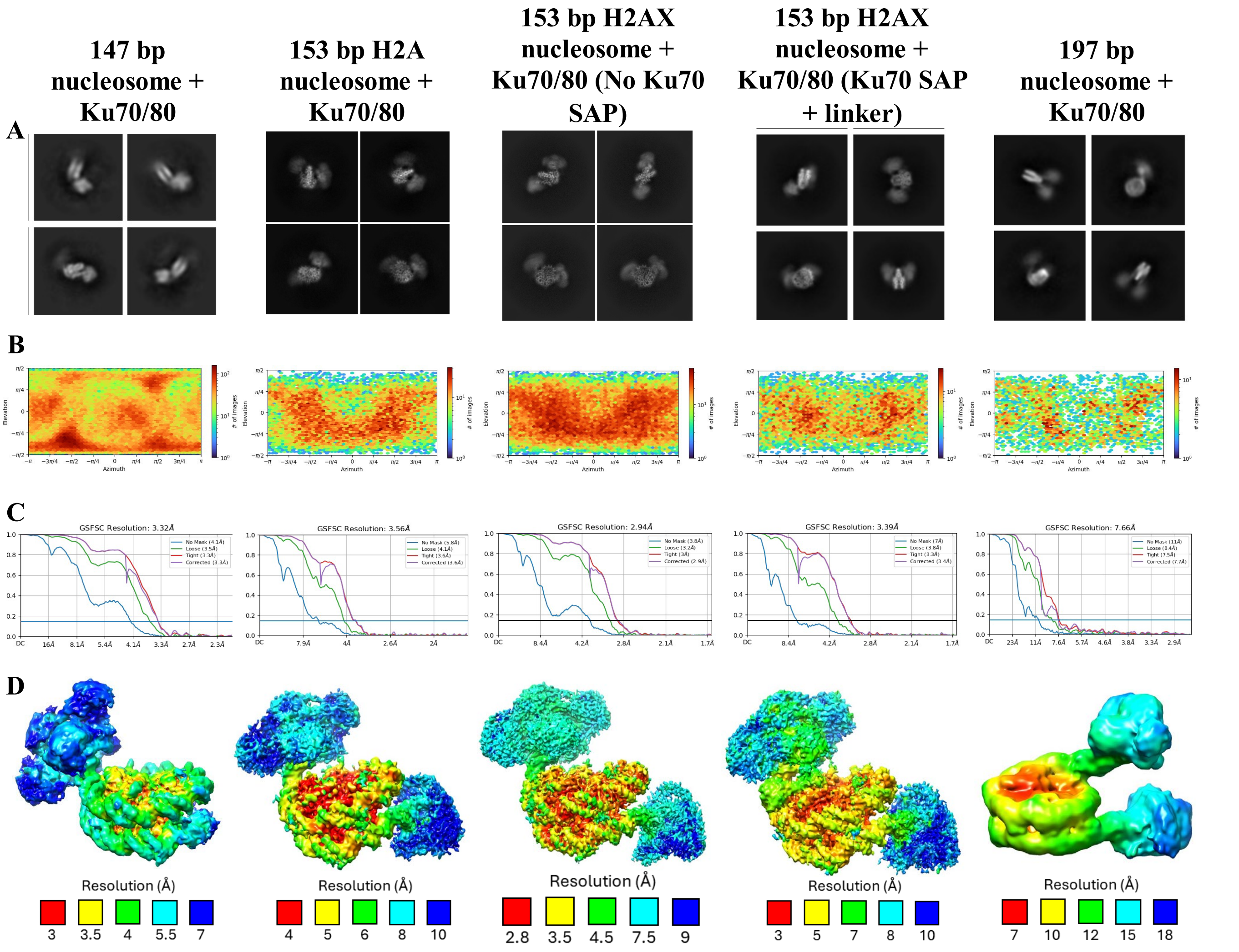

Supplementary Figure 6: Cryo-EM data for the Ku70/80 and nucleosome structures. A) representative 2D classes, **B)** viewing direction distribution for particle projections shown as heat maps generated by CryoSPARC, **C)** FSC resolution curves and **D)** Ku70/80 and nucleosome cryo-EM maps coloured by local resolution, the colours corresponding to each resolution are shown on the keys below.

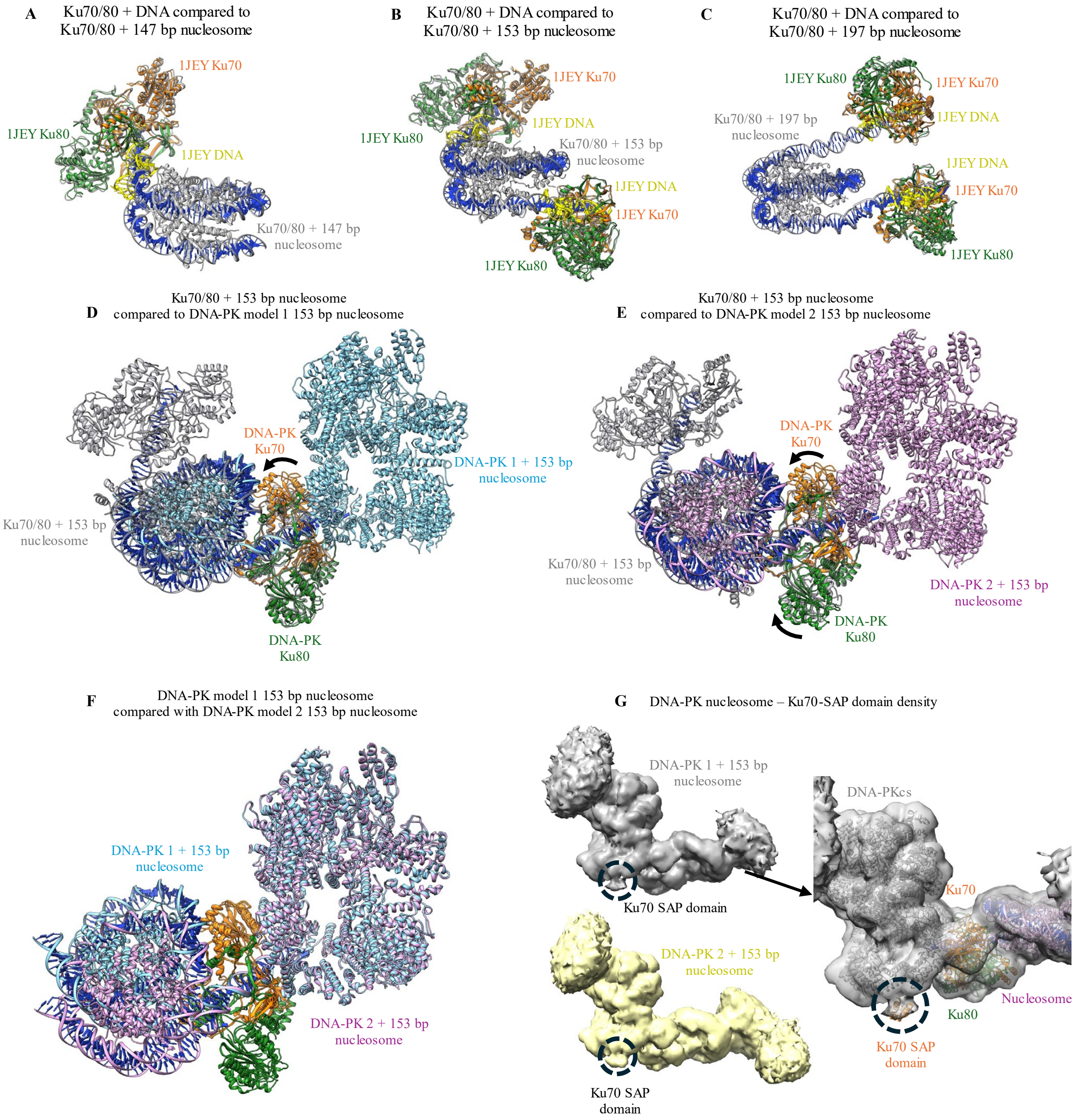

Supplementary Figure 7: Cryo-EM structural comparisons of the NHEJ machinery with nucleosomes. **A)** Ku70/80 + DNA (PDB: 1JEY) compared to Ku70/80 bound to a 147 bp nucleosome. **B)** Ku70/80 + DNA (PDB: 1JEY) compared to Ku70/80 bound to a 153 bp nucleosome. **C)** Ku70/80 + DNA (PDB: 1JEY) compared to Ku70/80 bound to a 197 bp nucleosome. 1JEY Ku70 is shown in orange, 1JEY Ku80 in green, 1JEY DNA in yellow and the nucleosome DNA and histones in grey. **D)** Ku70/80 with a 153 bp nucleosome (grey) compared to DNA-PK model 1 with a 153 bp nucleosome. DNA-PKcs is shown in blue, Ku70 orange, and Ku80 green from the DNA-PK model. **E)** Ku70/80 with a 153 bp nucleosome (grey) compared to DNA-PK model 1 with a 153 bp nucleosome. DNA-PKcs is shown in pink, Ku70 orange, and Ku80 green from the DNA-PK model 2. **F)** DNA-PK model 1 (blue) compared to DNA-PK model 2 (pink) with 153 bp nucleosomes with Ku70 in orange and Ku80 in green. **G)** DNA-PK nucleosome map model 1 (grey) and model 2 (yellow) showing where extra density for the Ku70 SAP domain on DNA-PKcs may reside.

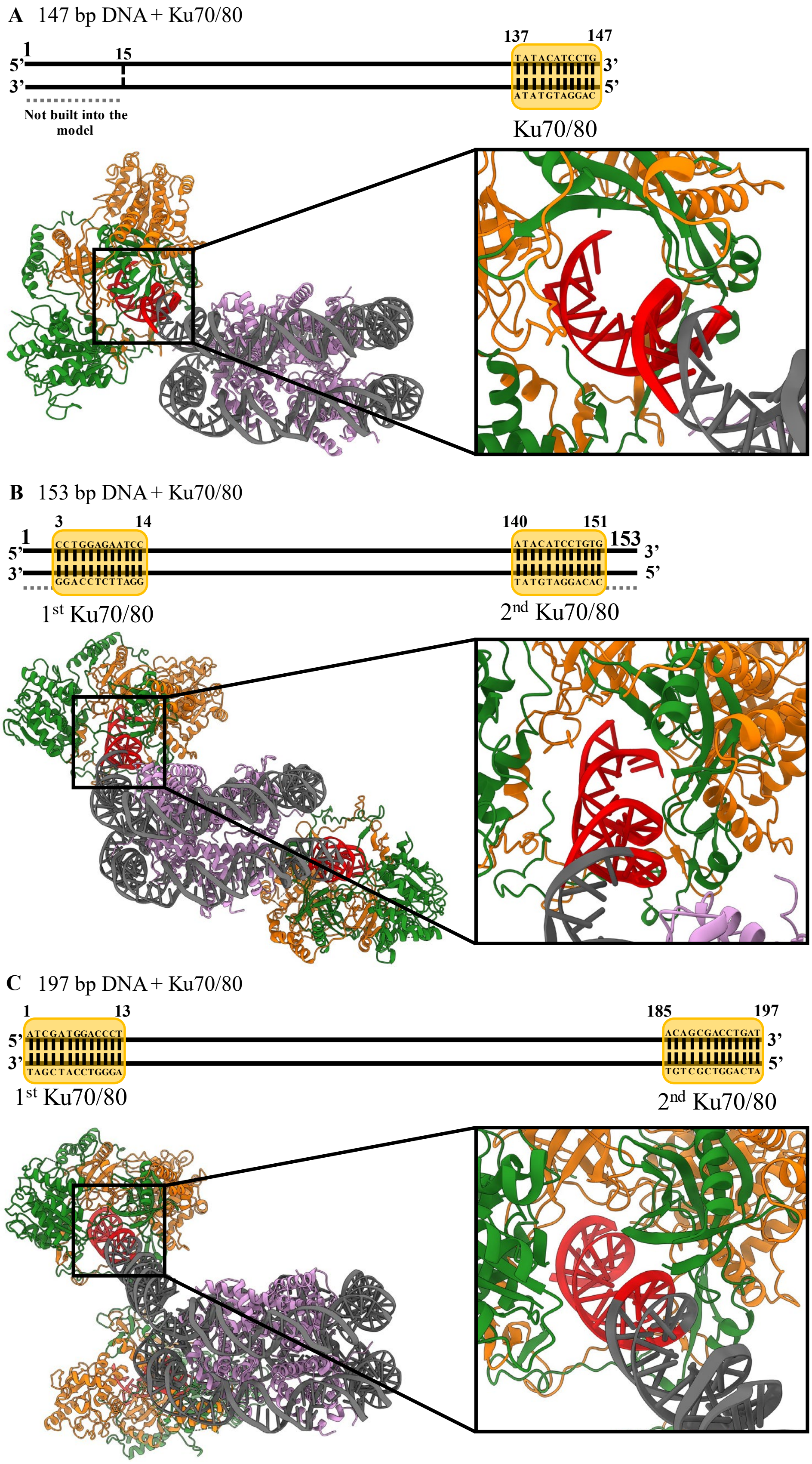

Supplementary Figure 8: DNA bases covered by Ku70/80 in the 147 bp, 153 bp and 197 bp nucleosome + Ku70/80 cryo-EM models. A) Ku70/80 bound to a 147 bp nucleosome cryo-EM model, DNA bases 137-147 (specific residues shown on the schematic and region of DNA highlighted in the inlet) are covered by Ku70/80. **B)** Ku70/80 bound to a 153 bp nucleosome cryo-EM model, DNA bases 3-14 and 140-151 are covered by the 1st and 2nd Ku70/80 molecules, respectively. **C)** Ku70/80 bound to a 197 bp nucleosome cryo-EM model, DNA bases 1-13 and 185-197 are covered by the 1st and 2nd Ku70/80 molecules, respectively. Ku70 coloured in orange, Ku80 in forest green, histones in pink and the nucleosome DNA in dim grey with the DNA bases covered by Ku70/80 in red. DNA coverage calculated using PISA analysis.

A 153 bp H2A DNA + Ku70/80

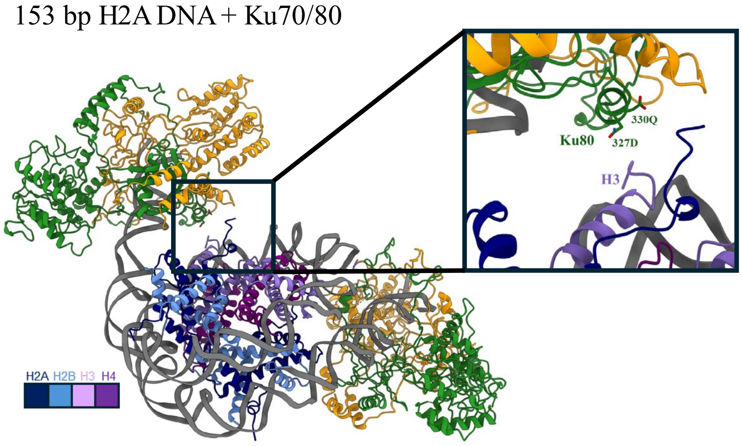

B Short-range complex (PDB: 7LSY)

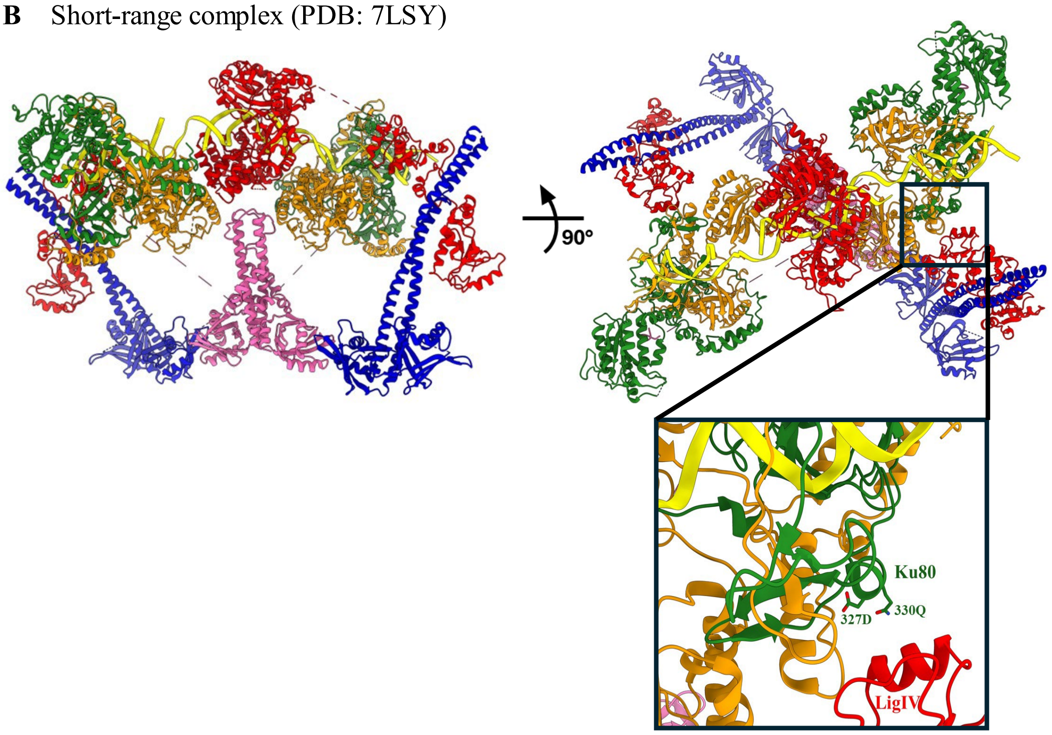

Supplementary Figure 9: Potential shared interaction interface between H3 and LigIV BRCT for Ku80. A) 153 bp H2A nucleosome and Ku70/80 model (this work), inlet highlighting potential interaction between H3 and Ku80. **B)** Cryo-EM structure of the short-range complex (PDB: 7LSY) in two different orientations, inlet highlighting the same region on Ku80 and LigIV BRCT interaction. Ku80 is shown in forest green, Ku70 orange, nucleosome DNA dim grey, histone colours shown in key, “free” DNA yellow, LigIV red, XRCC4 medium blue and XLF hot pink.

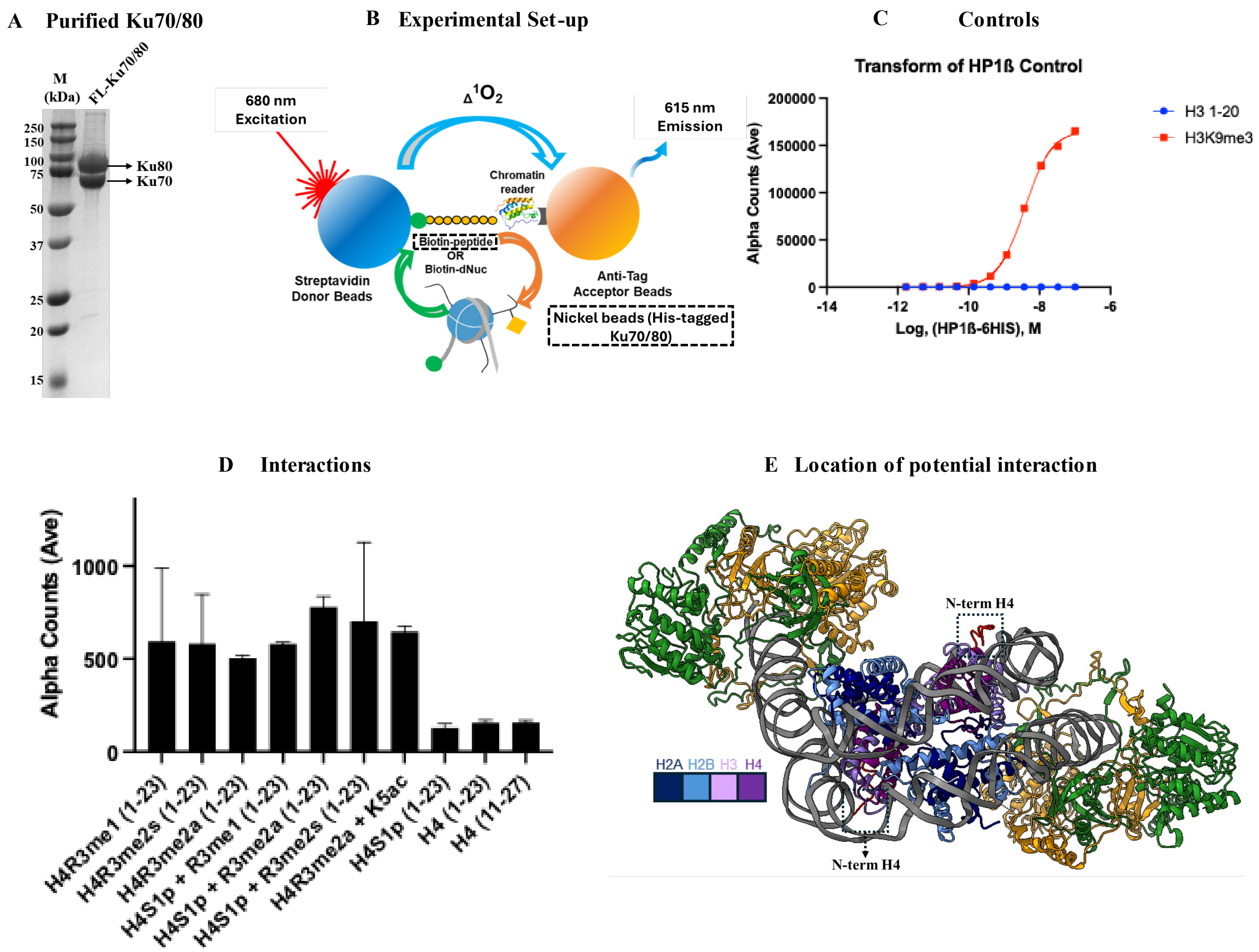

Supplementary Figure 10: EpiCypher results of Ku70/80 interacting with histone peptides. A) SDS-PAGE gel of purified Ku70/80. **B)** Experimental set-up for EpiCypher interactions between Ku70/80 and histone peptides, a single concentration of Ku70/80 was probed against 280 different biotinylated histone peptides. **C)** Controls performed as expected (HP1β). Overall, there was weak binding to the histone peptide as compared to the control. **D)** Interactions with counts and error bars. Potentially weak binding of Ku70/80 to H4R3me peptides from the peptide screen. **E)** Cryo-EM structure of Ku70/80 bound to 153bp nucleosome. Ku70 is shown in orange, Ku80 in forest green, DNA in dim grey, histone colours according to the key and the N-terminal of H4 is shown in red. There was no density corresponding to H4 residues 1-16, and so they were not built into the model; however, the N-terminal of H4 is not close to Ku70/80, and so an interaction is unlikely. The assay was performed with buffer (50 mM Tris pH 7.5 + 50 mM NaCl + 0.01% Tween-20 + 0.01% BSA + 1 mM TCEP + 0.0004% Poly-L-Lysine).

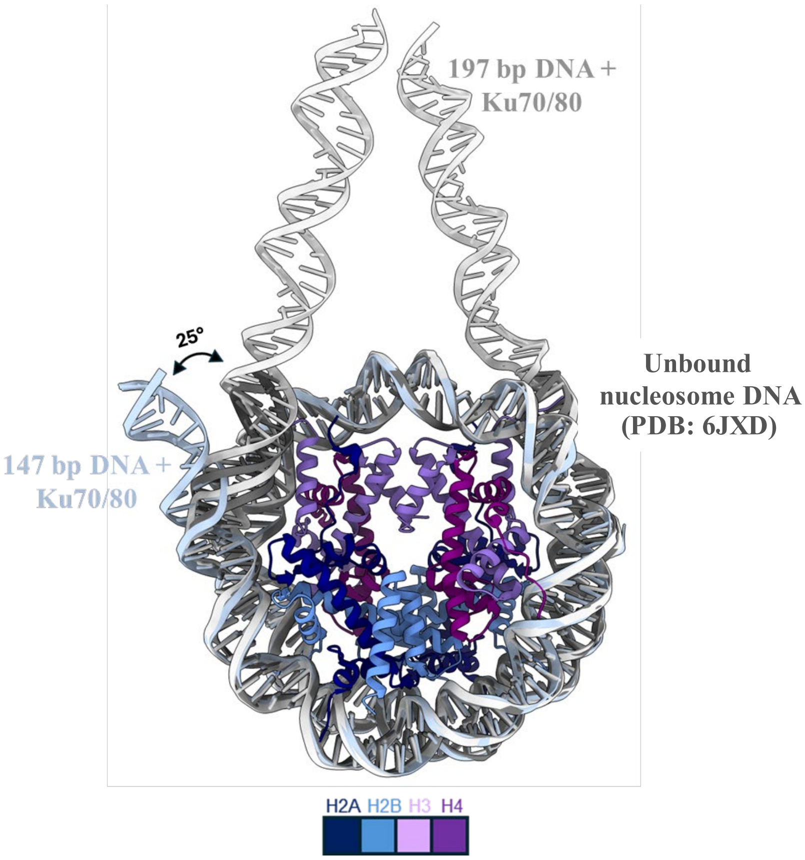

Supplementary Figure 11: DNA of the 147 bp nucleosome bound to Ku70/80 and the 197 bp nucleosome bound to Ku70/80 bending in comparison to an unbound nucleosome. The unbound nucleosome DNA (PDB: 6JXD) is shown in dim grey, the 197 bp DNA in silver, the 147 bp DNA in light steel blue, and histones coloured according to the key. The 197 bp DNA shows no bending when compared to the unbound nucleosome DNA, whereas the 147 bp DNA bends outwards approximately 25°.

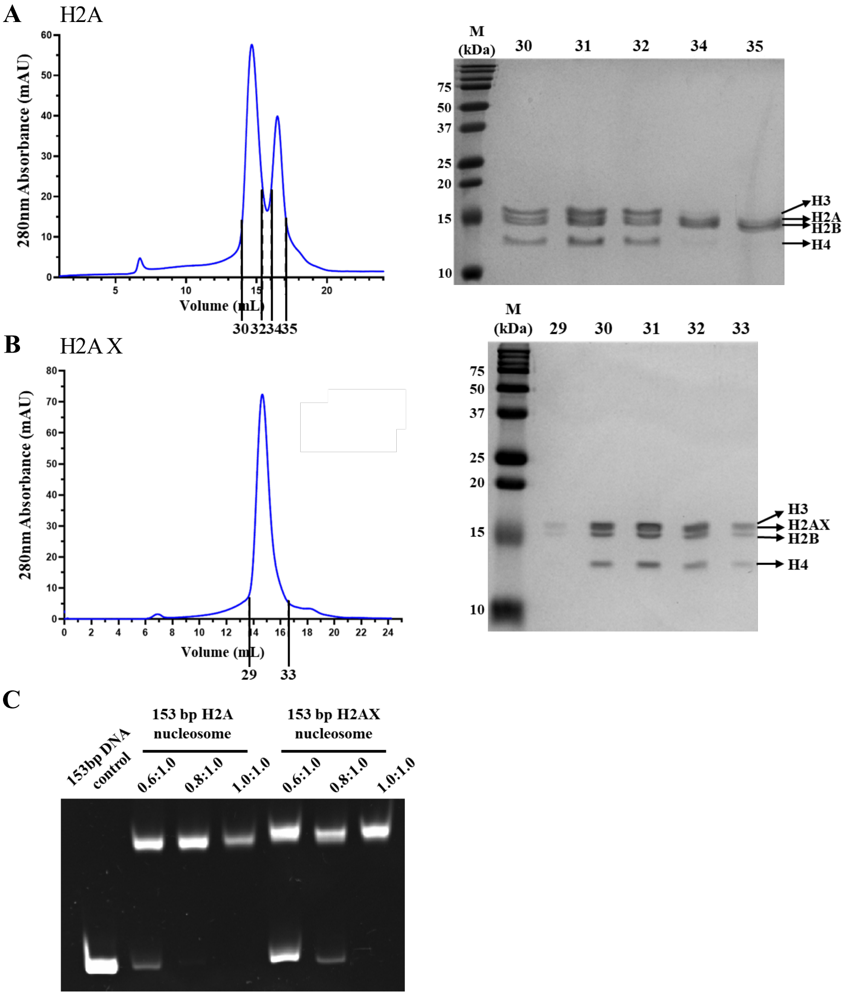

Supplementary Figure 12: 153 bp H2A and H2AX nucleosome reconstitution. A) H2A and **B)** H2AX superose 6 Increase 10/300 chromatogram for histone octamer purification (left) and corresponding 20% SDS-PAGE gel of the fractions spanning the 280nm Absorbance peak (right), bands corresponding to individual histones are labelled. **C)** 5% acrylamide native gel of the nucleosome reconstitution, the 153 bp DNA was run against increasing ratios of DNA: histone octamer to determine the optimal ratio to limit the amount of free DNA for both H2A and H2AX nucleosomes.

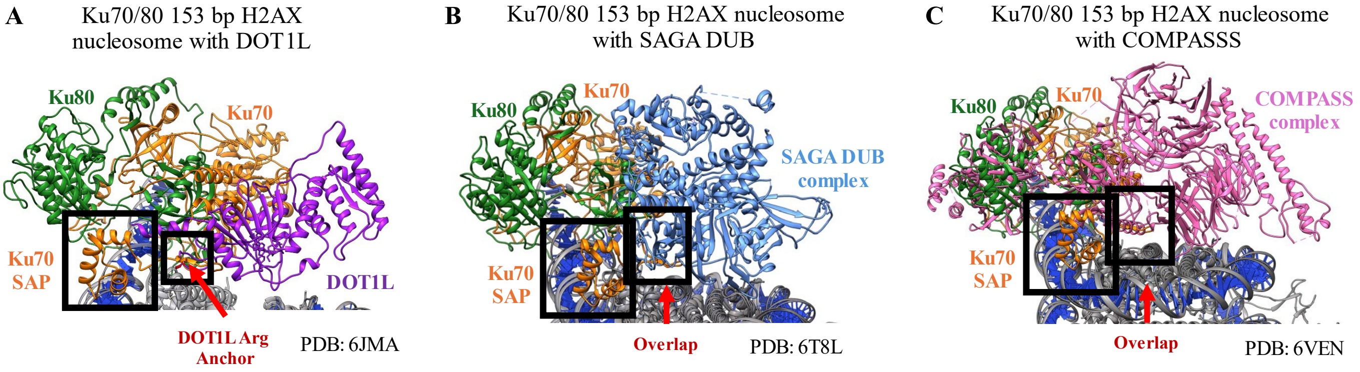

Supplementary Figure 13: A comparison of Ku70 SAP domain bound to nucleosomes compared to DOT1L, SAGA DUB complex and COMPASS complex. A) Ku70/80 153bp H2AX nucleosome compared to DOT1L. Ku70 in orange, Ku80 in green, DOT1L in purple and DOT1L Arg anchor shown in red. **B)** Ku70/80 153 bp H2AX nucleosome with SAGA DUB. Ku70 in orange, Ku80 in green, SAGA DUB in blue. **C)** Ku70/80 153 bp H2AX nucleosome with COMPASS. Ku70 in orange, Ku80 in green, COMPASS in pink.

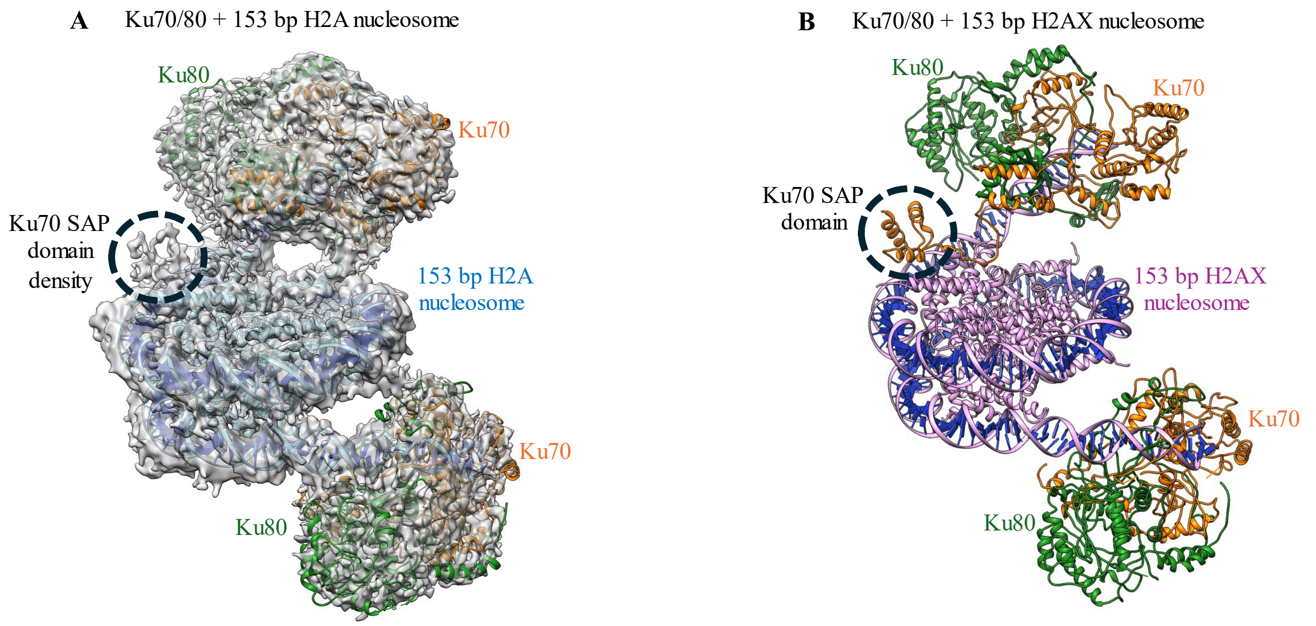

Supplementary Figure 14: Ku70 SAP domain on nucleosomes. A) Ku70/80 bound to a 153 bp nucleosome containing H2A map and model superimposed. Extra density corresponding to the Ku70 SAP domain is circled. Ku70 is shown in orange, Ku80 in forest green and the nucleosome in blue. **B)** Ku70/80 bound to a 153 bp nucleosome containing H2AX histone model, Ku70 SAP domain is circled. Ku70 is shown in orange, Ku80 in forest green and the nucleosome in pink.

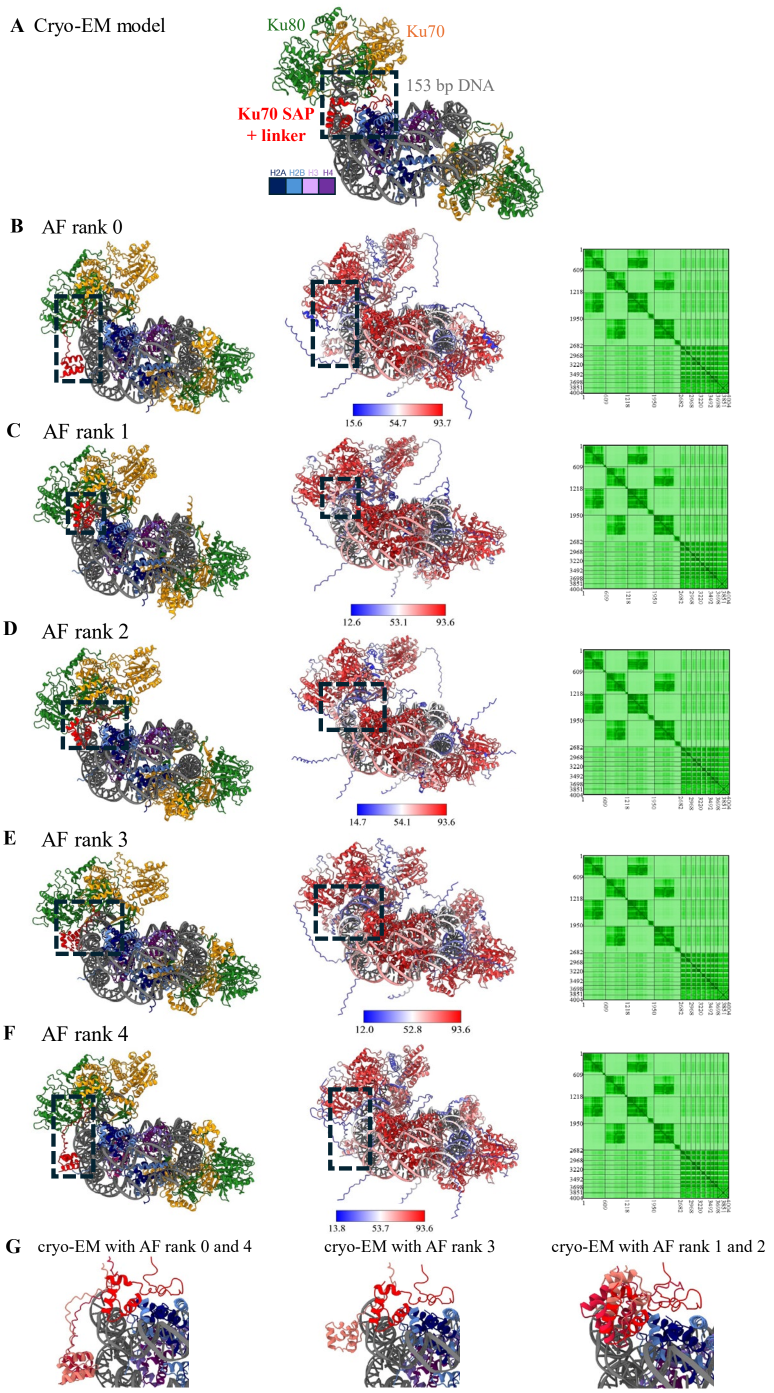

Supplementary Figure 15: AlphaFold (AF) 3 of a 153 bp nucleosome containing H2AX with 2x copies of full length-Ku70/80. Two copies of full-length human Ku80 and Ku70, two copies of each full-length histone H2AX, H2B, H3 and H4 and 153 bp DNA were put into the AF 3 server (Abramson *et al.,* 2024). **A)** The obtained experimental cryo-EM model of Ku70/80 bound to a 153 bp nucleosome containing H2AX. **B, C, D, E and F)** AlphaFold ranks in corresponding order rank 0 to rank 4, with the AF model presented on the left, for direct comparison with the cryo-EM model the Ku80 c-terminus is hidden (residues 543-732) and the N and C terminus of each histone that were not built into the cryo-EM model are also hidden, shown are H2AX (residues 11-119), H2B (residues 26-122), H3 (residues 39-133), H4 (residues 23-100). Ku70 is shown in orange with the SAP domain and linker region in red, Ku80 in forest green, nucleosome DNA in dim grey, H2AX in navy, H2B in cornflower blue, H3 in medium purple, and H4 in purple. The middle panel shows each AF model coloured based on confidence scoring (coloured by b-factor in ChimeraX), the colour keys are shown below (blue being least confident and red being most confident). The right panel shows each PAE plot. **G)** The experimental model superimposed with the AF ranks, the cryo-EM model SAP and linker regions are coloured in red, and the AF ranks are coloured in salmon (rank 0, 1 and 3) and crimson (rank 2 and 4). For comparisons, the cryo-EM models and AF ranks were aligned based on the nucleosome DNA using the MatchMaker tool in UCSF ChimeraX.

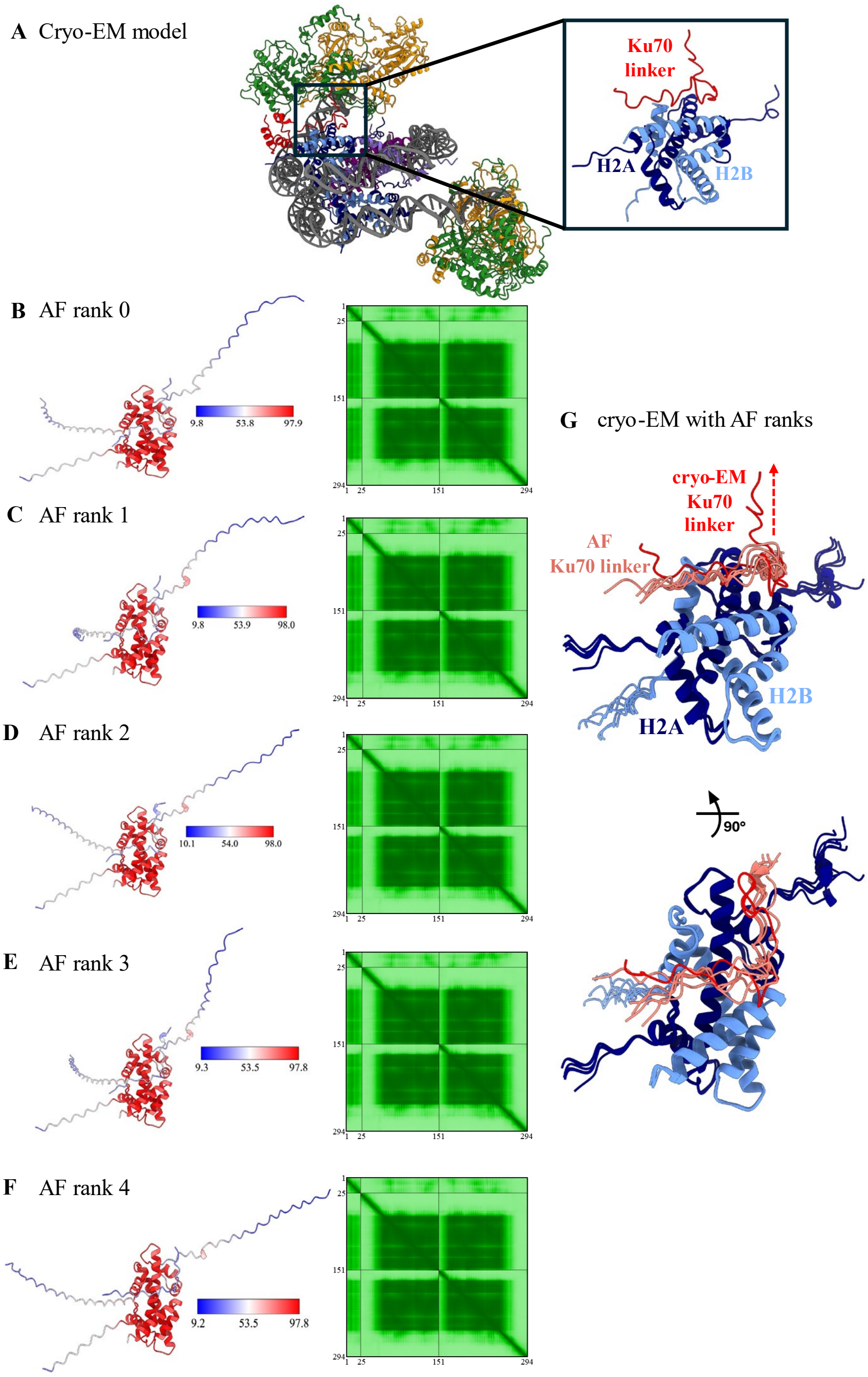

Supplementary Figure 16: AF 3 of H2AX and H2B with the Ku70 linker (residues 535-559). One copy of full-length histones H2AX and H2B, and the Ku70 residues 535-559, were put into the AF 3 server (Abramson *et al.,* 2024). **A)** The obtained experimental cryo-EM model of Ku70/80 bound to a 153 bp nucleosome containing H2AX with an inlet highlighting the H2AX H2B Ku70 linker region. **B, C, D, E and F)** The AF ranks in corresponding order rank 0 to rank 4, with the AF model presented on the left coloured based on confidence (b-factor colouring in UCSF ChimeraX), coloured according to the key (blue the least confident and red most confident). The middle panel shows the PAE plots. **G)** The cryo-EM model superimposed with the AF ranks in two different orientations, aligned using the MatchMaker tool in ChimeraX, for direct comparison, the C-terminal tails of H2AX and H2B for each AF model are hidden. The experimental cryo-EM Ku70 linker is coloured in red, and the AF Ku70 linker in salmon, H2B in cornflower blue and H2AXin navy.

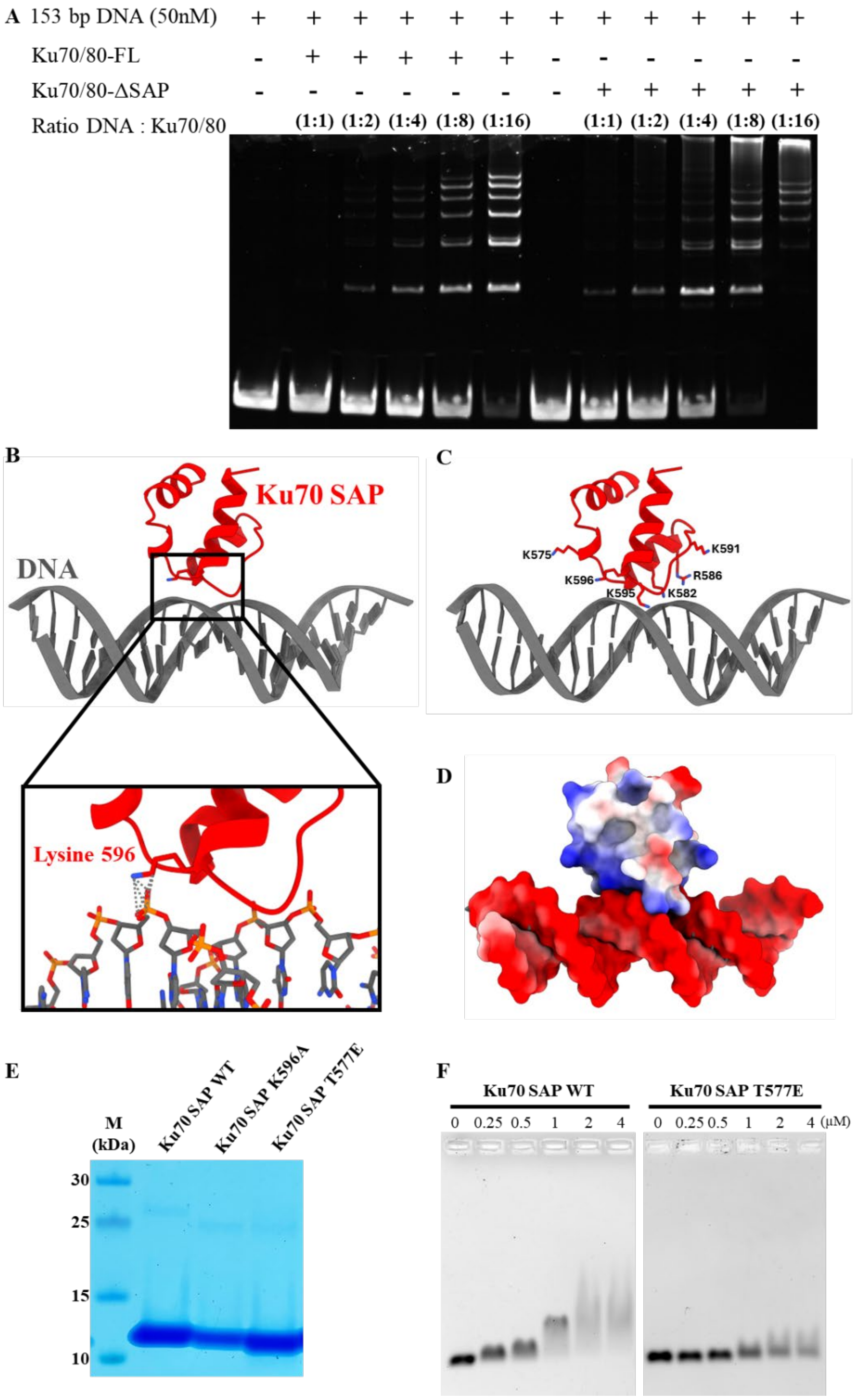

Supplementary Figure 17: Full-length Ku70/80 vS Ku70/80 ΔSAP binding to 153 bp DNA EMSA, AF of Ku70 SAP domain with DNA and Ku70 SAP WT vS Ku70 SAP T577E EMSA. A) FL-Ku70/80 and Ku70/80-ΔSAP against 153 bp DNA EMSA. The DNA concentration was kept constant (50nM), and Ku70/80 (FL or ΔSAP) was titrated at varying ratios displayed. B) One copy of Ku70 SAP domain (residues 559-609) and a 20bp DNA (sequence: 5' ATCCTGGAGAATCCCGGTGC 3') were put into the AF 3 server (Abramson *et al.,* 2024). AF rank 0 is presented, Ku70 SAP domain in red and DNA in dim grey. Inlet showing Ku70 SAP lysine 596 interacting with the DNA. C) AF model with additional positively charged residues in proximity to DNA highlighted. D) AF model coloured by electrostatic charge (red and blue being negatively and positively charged, respectively). E) Coomassie-stained gel after denaturing and reducing SDS-PAGE of the purified Ku70 SAP domain residues 539-609 (WT, K596A, and T577E). F) EMSA detecting retardation of a 400 bp DNA fragment by purified WT or T577E Ku70 SAP domain in isolation (residues 539-609), indicative of a direct interaction with DNA stabilized by residue T577.

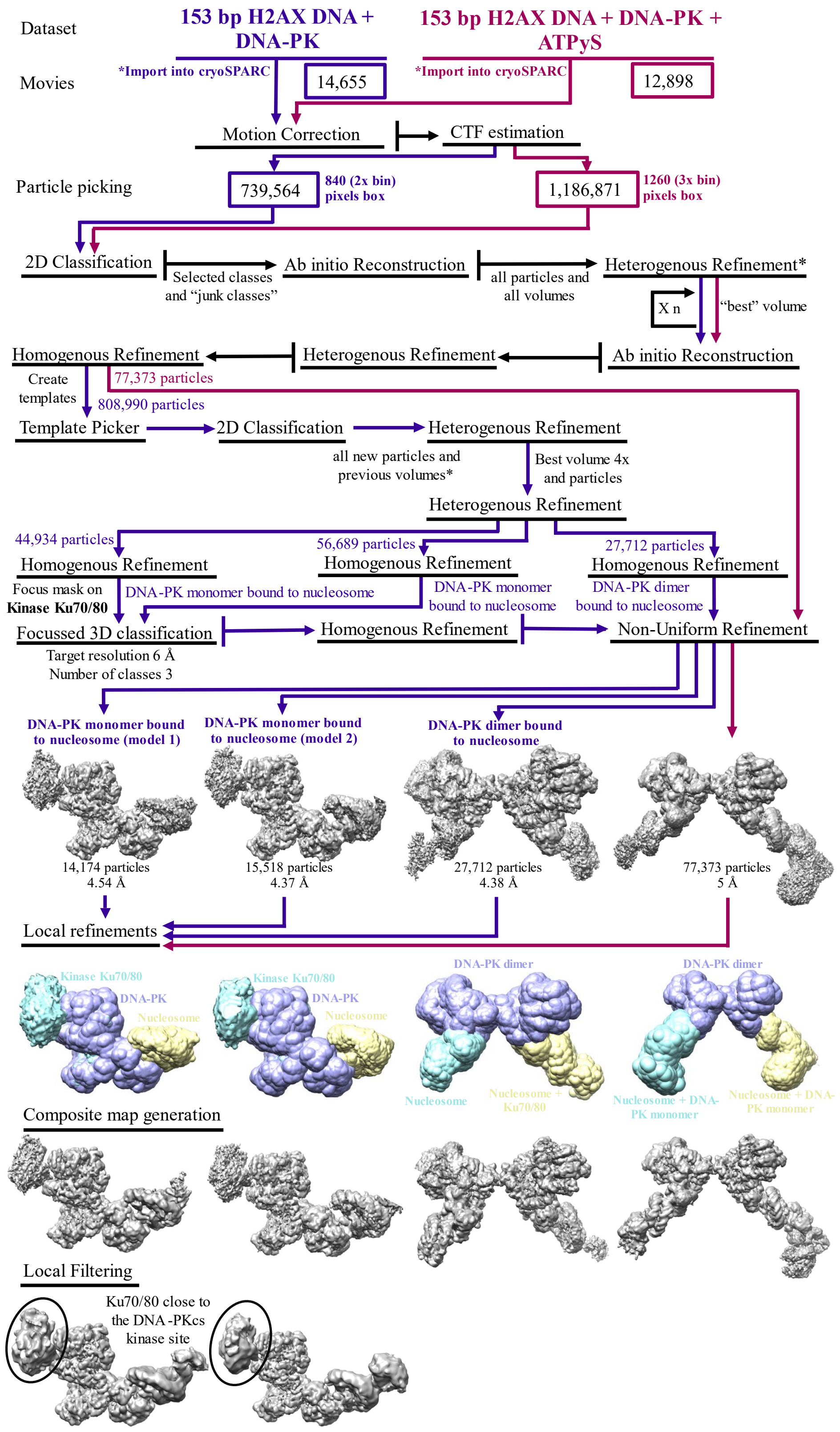

Supplementary Figure 18: Single particle cryo-EM data processing workflows for the DNA-PK and nucleosome maps. For both 153 bp H2AX nucleosome and DNA-PK datasets the whole data processing workflow was completed in CryoSPARC. The picked particles for both datasets were subject to 2D classification, and from this, the "good" and "junk" classes were used to generate 3D models via *ab initio reconstruction.* A heterogeneous refinement was then completed with all particles and volumes. Following this, varying refinements and classifications were completed to separate out the distinct maps from the datasets. The final maps were subject to non-uniform refinement; the number of particles and resolution for an FSC of 0.143 is shown. For all the final refined maps, local regions were refined to improve the density in certain areas, and these maps were used to generate composite maps. For both DNA-PK monomer bound to a nucleosome maps local filtering was completed to better visualise the kinase Ku70/80.

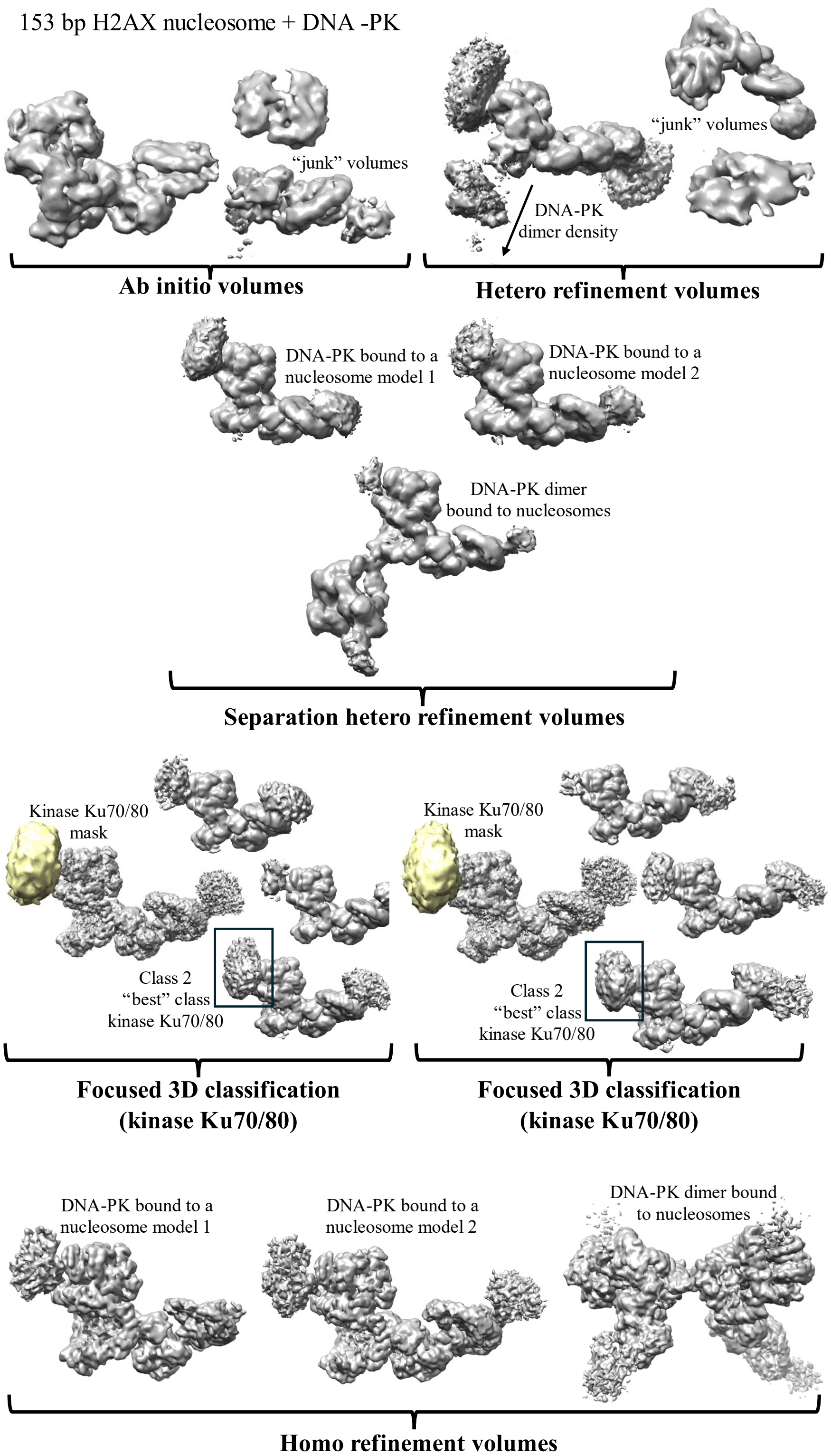

Supplementary Figure 19: Intermediate maps from data processing of the 153 bp H2AX nucleosome and DNA-PK. Volumes from *ab initio reconstruction*, heterogeneous refinements, focused 3D classification and homogenous refinement are shown, including "junk" volumes.

153 bp H2AX nucleosome + DNA -PK + ATPyS

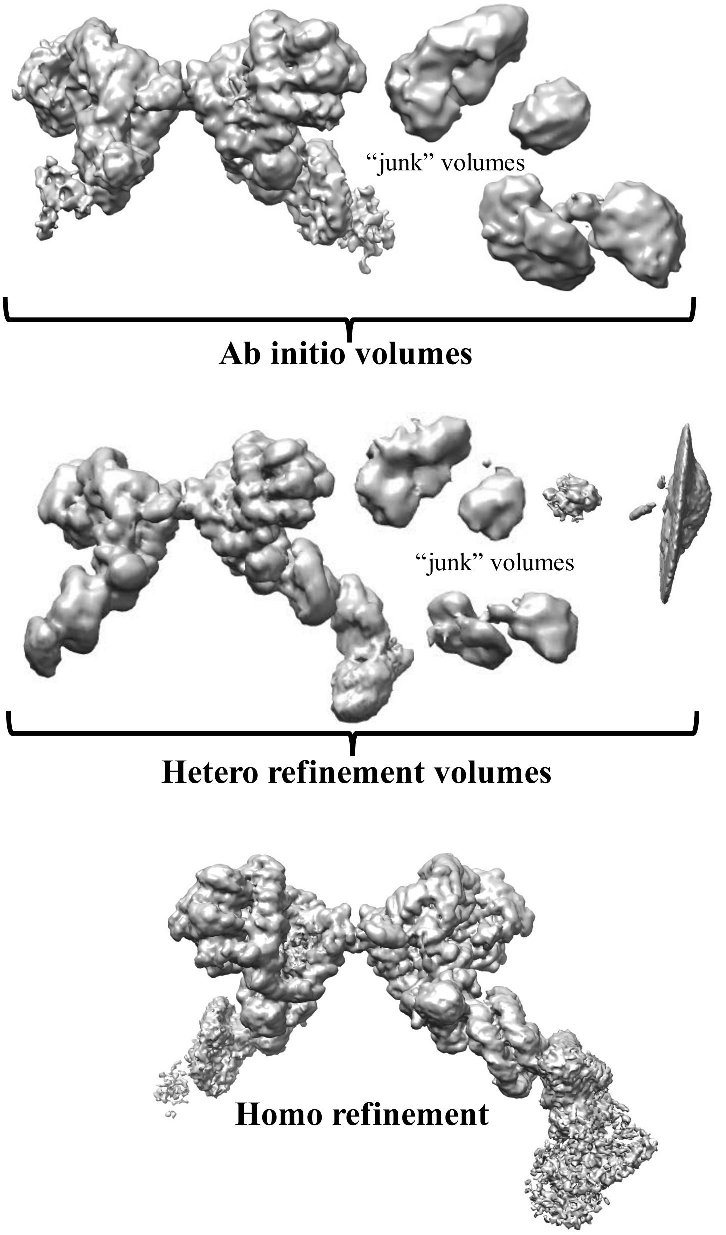

Supplementary Figure 20: Intermediate maps from data processing of the 153 bp H2AX nucleosome and DNA-PK with ATPyS. Volumes from *ab initio reconstruction*, heterogeneous refinement, and homogenous refinement are shown, including "junk" volumes.

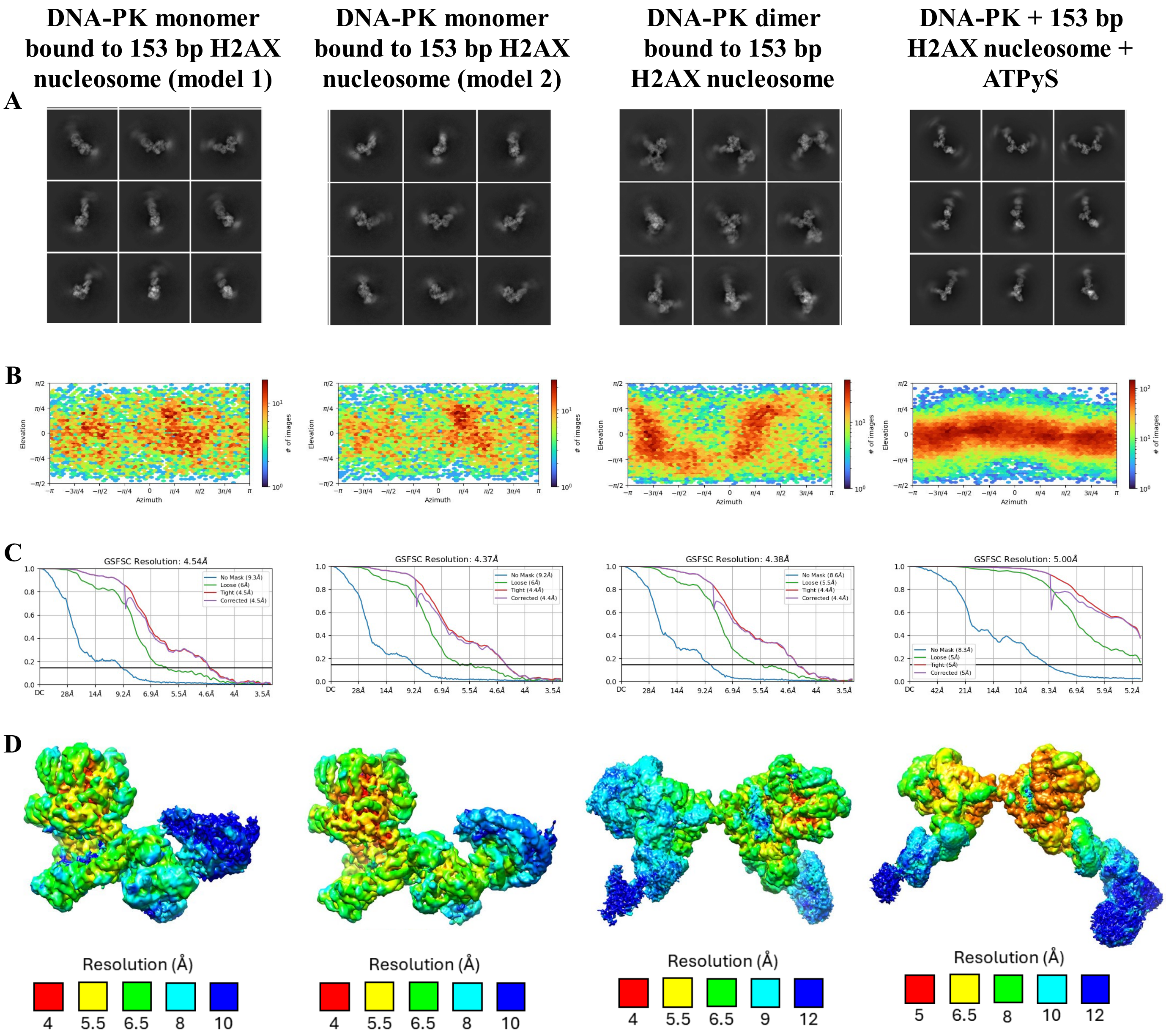

Supplementary Figure 21: Cryo-EM data for the DNA-PK and nucleosome structures. A) representative 2D classes, **B)** viewing direction distribution for particle projections shown as heat maps generated by CryoSPARC, **C)** FSC resolution curves and **D)** DNA-PK and nucleosome cryo-EM maps coloured by local resolution; the colours corresponding to each resolution are shown on the keys below.

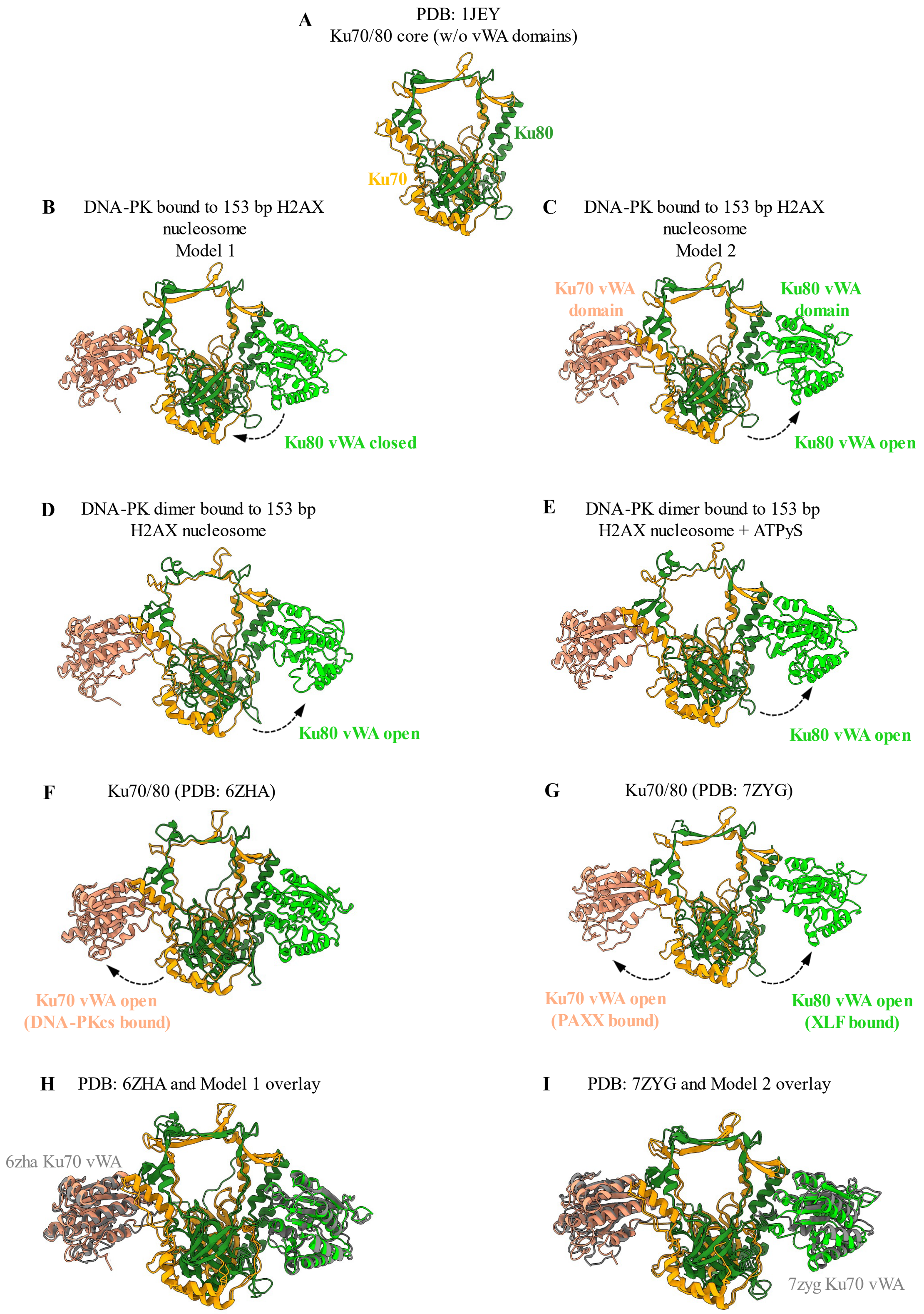

Supplementary Figure 22: Comparisons of the Ku70/80 vWA opening for the DNA-PK bound to a 153 bp H2AX nucleosome maps. A) Ku70/80 (PDB: 1JEY) with Ku70 and Ku80 vWA domains deleted, used as the reference structure for model alignments using the MatchMaker tool in Chimera. **B and C)** DNA-PK monomer bound to the 153 bp H2AX nucleosome models, with the Ku80 vWA domain closed and open in models 1 and 2, respectively. **D and E)** DNA-PK dimer bound to the 153 bp H2AX nucleosome models with and without ATPγS, respectively. **F)** DNA-PK (PDB: 6ZHA), where Ku70 vWA is open when DNA-PKcs is bound). **G)** Ku70/80 bound to XLF and PAXX (PDB: 7ZYG), where both Ku70 and Ku80 vWA's are open. **H and I)** overlay of PDB: 6ZHA with DNA-PK model 1 and PDB: 7ZYG with DNA-PK model 2, respectively, 6ZHA and 7ZYG Ku vWA domains coloured grey for comparison purposes. Ku70 core shown in orange with the vWA domain in light salmon, and Ku80 core forest green with the vWA domain in lime.

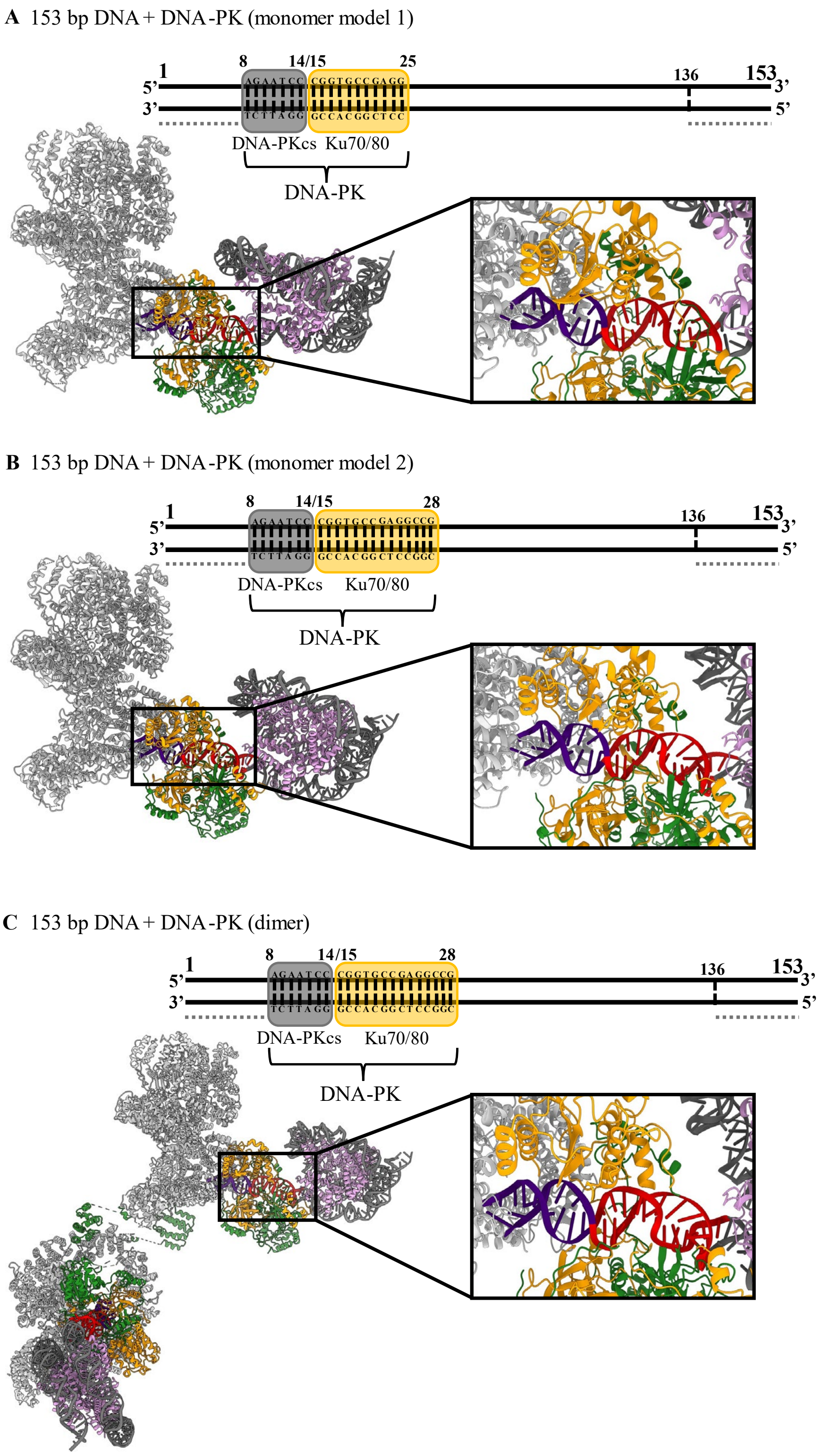

Supplementary Figure 23: DNA bases covered by Ku70/80 and DNA-PKcs in the 153 bp nucleosome bound to DNA-PK models. A) DNA-PK monomer bound to a 153 bp nucleosome cryo-EM model 1, DNA bases 8-14 and 15-25 (specific residues shown on the schematic and region of DNA highlighted in the inlet) are covered by DNA-PKcs and Ku70/80, respectively. **B)** DNA-PK monomer bound to a 153 bp nucleosome cryo-EM model 2, DNA bases 8-14 and 15-28 are covered by DNA-PKcs and Ku70/80, respectively. **C)** DNA-PK dimer bound to a 153 bp nucleosome cryo-EM model, DNA bases 8-14 and 15-28 are covered by DNA-PKcs and Ku70/80, respectively. DNA-PKcs coloured in grey, Ku70 in orange, Ku80 in forest green, histones in pink and the nucleosome DNA in dim grey with the DNA bases covered by Ku70/80 in red and DNA-PKcs in purple. DNA coverage calculated using PISA analysis.

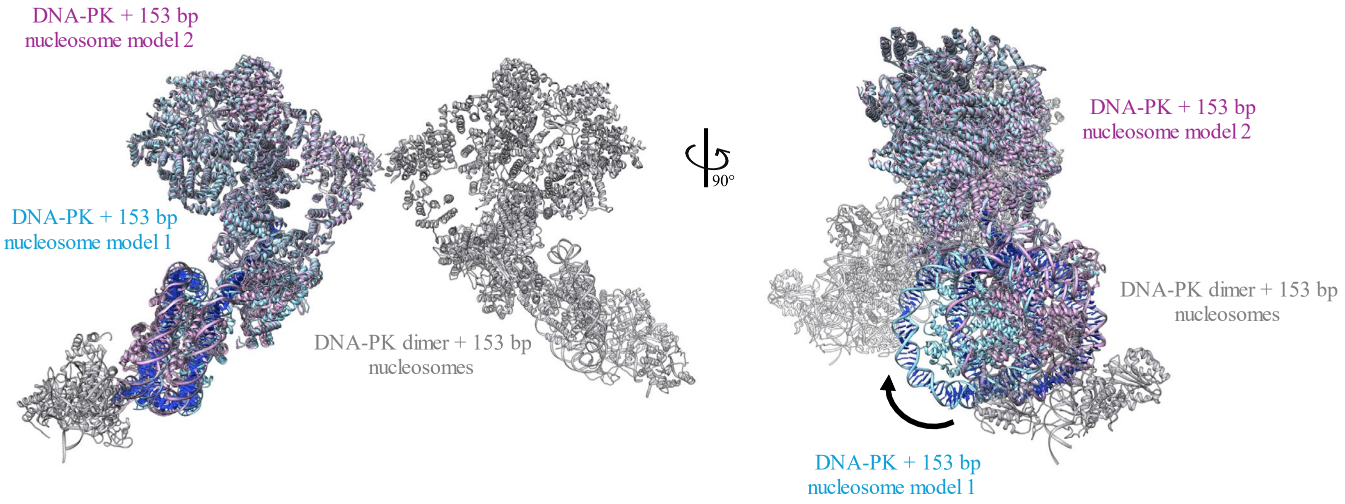

Supplementary Figure 24: A comparison of DNA-PK bound to nucleosomes. DNA-PK dimer bound to 153 bp nucleosomes shown in grey, compared to DNA-PK bound to a 153 bp nucleosome model 1 in blue and model 2 in pink.

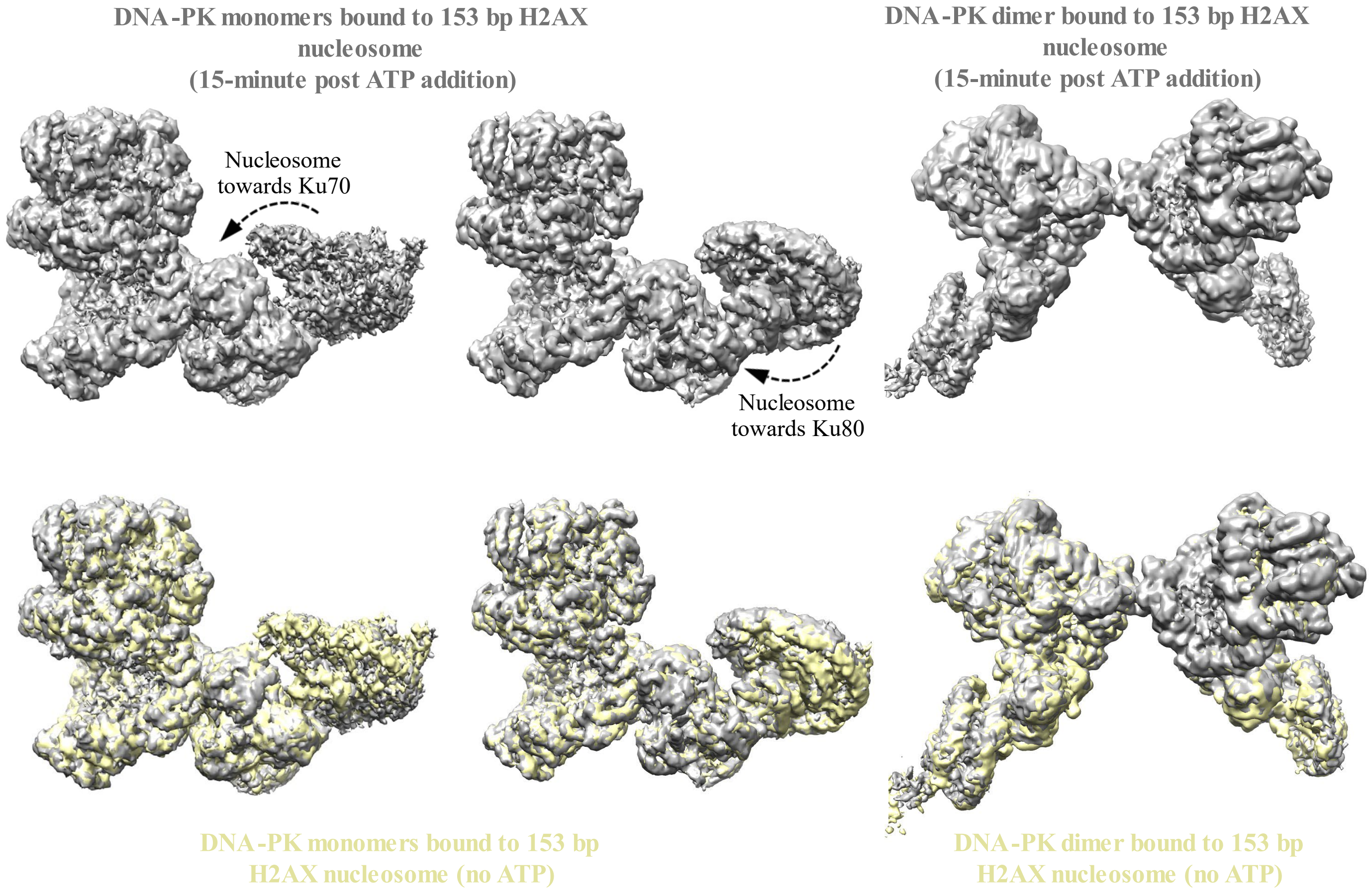

Supplementary Figure 25: DNA-PK bound to a 153 bp H2AX nucleosome + ATP density maps. The top panel shows three distinct density maps from a dataset of 153 bp H2AX nucleosome with DNA-PK and ATP (added 15 minutes before sample vitrification). The bottom panel shows the density maps collected and processed from the dataset with ATP (grey) superimposed with the density maps without ATP (yellow). Highlighting that there is no difference between the maps produced from each dataset.

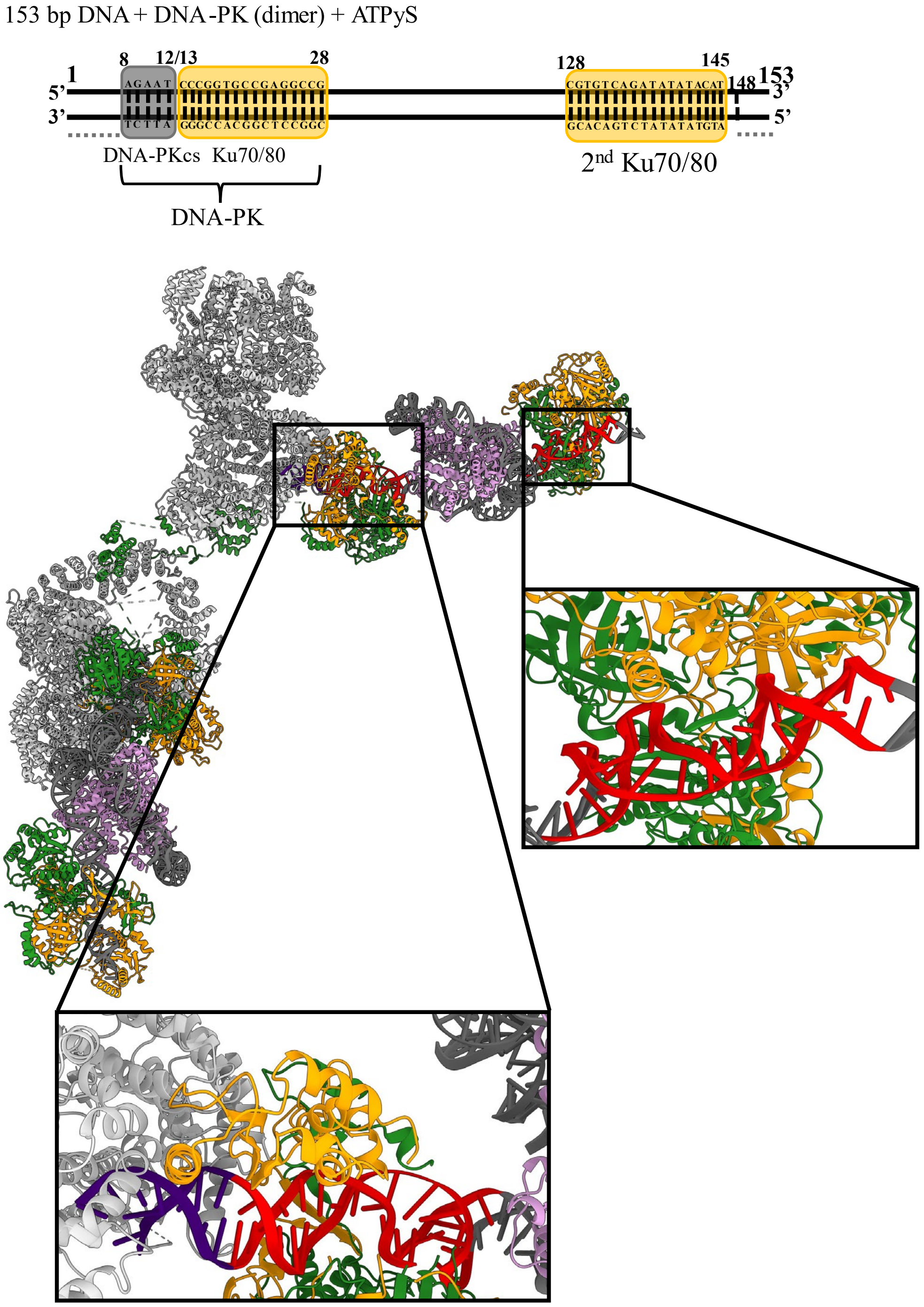

Supplementary Figure 26: DNA bases covered by Ku70/80 and DNA-PKcs in the 153 bp nucleosome bound to DNA-PK with ATPyS model. DNA-PK dimer bound to a 153 bp nucleosome with ATPyS cryo-EM model, DNA bases 8-12 and 13-28 are covered by DNA-PKcs and Ku70/80, respectively, and DNA bases 128-145 are covered by a 2nd molecule of Ku70/80 (specific residues shown on the schematic and regions of DNA highlighted in the inlets). DNA-PKcs coloured in grey, Ku70 in orange, Ku80 in forest green, histones in pink and the nucleosome DNA in dim grey with the DNA bases covered by Ku70/80 in red and DNA-PKcs in purple. DNA coverage calculated using PISA analysis.

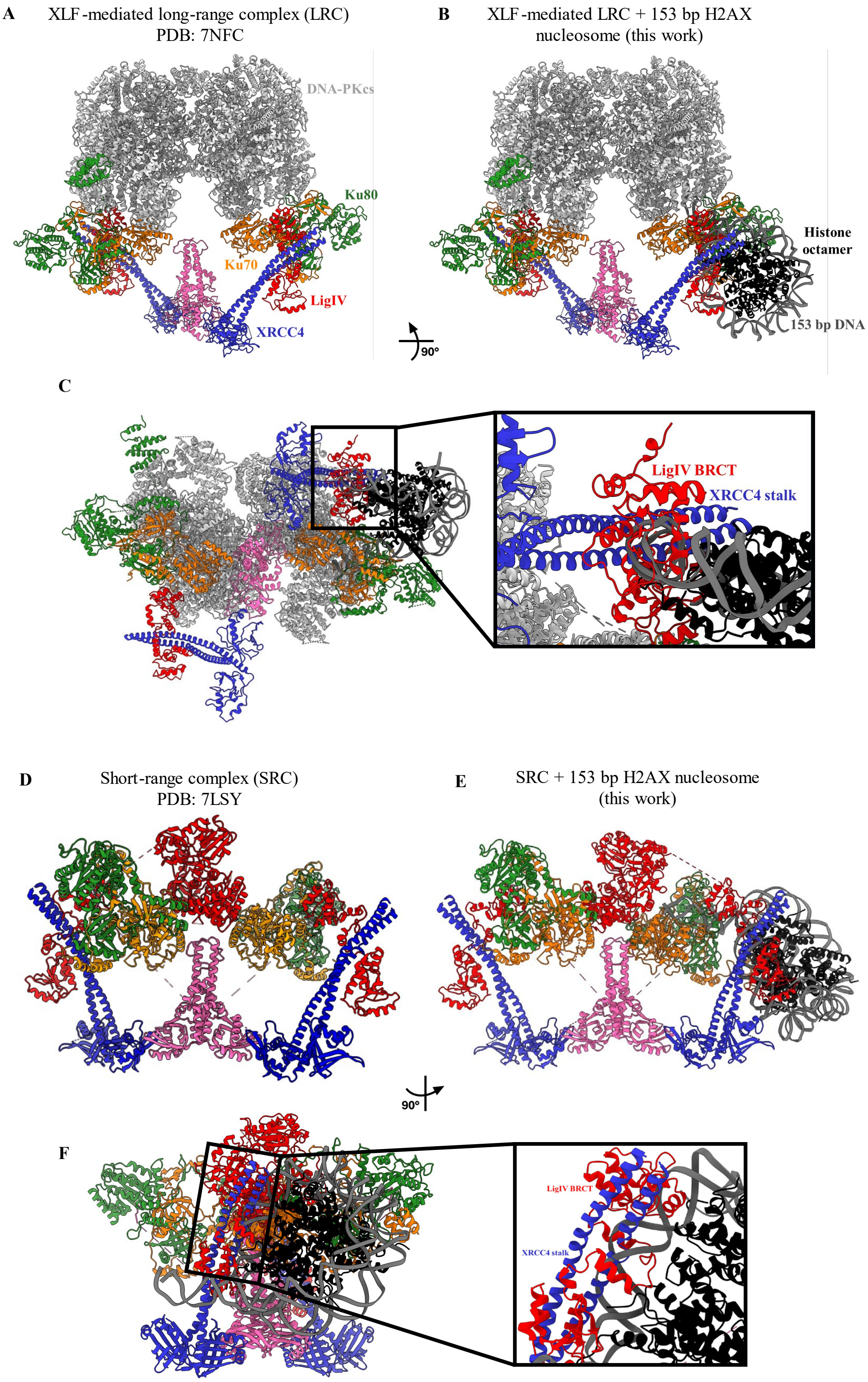

Supplementary Figure 27: XLF-mediated LRC and short-range complex clashing with the Ku70/80 bound to a 153 bp H2AX nucleosome model. A and D) The XLF-mediated LRC and SRC (PDB: 7NFC and 7LSY, with the free DNA chains removed), respectively. **B and E)** Ku70/80 in the LRC and SRC were aligned with the Ku70/80 from the 153 bp H2AX nucleosome and Ku70/80 model (this work). **C and F)** inlets to show the steric clashes between the nucleosome DNA and the LigIV BRCT domains and the stalk of XRCC4. DNA-PKcs coloured in grey, Ku80 forest green, Ku70 orange, LigIV red, XRCC4 medium blue, XLF hot pink, nucleosome DNA dim grey and the histone octamer in black.

Supplementary table 1:
Cryo-EM data collection and refinement statistics for Ku70/80 bound to nucleosome models.

	Ku70/80 + 147bp nucleosome<br>PDB: 9IGW<br>EMDB: 52860	Ku70/80 + 153bp (H2A) nucleosome<br>PDB: 9IGX<br>EMDB: 52861	Ku70/80 + 153bp H2AX nucleosome SAP<br>PDB: 9Q80<br>EMDB: 52879	Ku70/80 + 153bp H2AX nucleosome NO SAP<br>PDB: 9Q8X<br>EMDB: 52912	Ku70/80 + 197bp nucleosome
**Data collection and processing:**					
Microscope	Titan Krios	Titian Krios	Titian Krios	Titian Krios	Titan Krios
Detector	Gatan K3	Gatan K3	Gatan K3	Gatan K3	Falcon III
Magnification	81k	105k	105k	105k	120k
Energy filter slit width (eV)	20	20	20	20	20
Voltage (kV)	300	300	300	300	200
Flux on the detector (e/pix/sec)	17.572	17.453	16.1	16.1	1.3
Electron exposure on the sample (e-/Å ^{2})	40.98	50.01	50.1	50.1	51.84
Defocus range (µm)	-2.4 to -0.9	-2.2 to -0.7	-2.2 to -0.7	-2.2 to -0.7	-1.4 to 3.0
Calibrated pixel range (Å)	1.07	0.824	0.824	0.824	0.650
Symmetry imposed	C1	C1	C1	C1	C1
Extraction box size (pixels)	380	480	480	480	352 (2x bin)
Initial particle images (no.)	1091688	2596956	2328334	2328334	211476
Final particle images (no.)	103085	24500	16914	48185	8822
Map resolution at FSC=0.143 (Å)	3.32 Å	3.56Å	3.39 Å	2.94Å	7.66 Å
**Refinement:**					
Model composition					

Non-hydrogen atoms	18919	27730	27777	27522	-
Protein residues	1778	2797	2877	2797	-
Nucleotides	264	298	299	300	-
B factor (Å^2)					
Protein	413.08	605.21	619.67	296.77	-
DNA	182.89	222.58	261.19	143.22	-
R.m.s deviations					
Bond lengths (Å)	0.004	0.003	0.003	0.003	-
Bond angles (°)	0.616	0.580	0.593	0.600	-
Validation					
Molprobity score	2.04	2.15	2.15	1.99	-
Clash score	17.36	17.25	10.72	12.15	-
Poor rotamers (%)	0.29	0.52	1.14	0.59	-
Ramachandran plot					
Favoured (%)	95.61	93.76	89.53	94.17	-
Allowed (%)	4.33	6.17	10.16	5.69	-
Disallowed (%)	0.06	0.07	0.32	0.14	-

Supplementary table 2:
Cryo-EM data collection and refinement statistics for DNA-PK bound to nucleosome models.

	DNA-PK + 153bp H2AX nucleosome (model 1)<br>PDB: 9Q9F<br>EMDB: 52958	DNA-PK + 153bp H2AX nucleosome (model 2)<br>PDB: 9QCR<br>EMDB: 53025	Ku80 mediated DNA-PK dimer + 153bp H2AX nucleosome<br>PDB: 9QCS<br>EMDB: 53026	Ku80 mediated DNA-PK dimer + 153bp H2AX nucleosome + ATPyS<br>PDB: 9QMS<br>EMDB: 53237
**Data collection and processing:**				
Microscope	Titan Krios	Titian Krios	Titian Krios	Titian Krios
Detector	Gatan K3	Gatan K3	Gatan K3	Gatan K3
Magnification	105k	105k	105k	105k
Energy filter slit width (eV)	20	20	20	20
Voltage (kV)	300	300	300	300
Flux on the detector (e/pix/sec)	15.4	15.4	15.4	15.7
Electron exposure on the sample (e-/Å ^{2})	48.59	48.59	48.59	50.87
Defocus range (µm)	-2.2 to -0.7	-2.2 to -0.7	-2.2 to -0.7	-2.0 to -0.8
Calibrated pixel range (Å)	0.824	0.824	0.824	0.824
Symmetry imposed	C1	C1	C1	C1
Extraction box size (pixels)	840 (2x bin)	840 (2x bin)	840 (2x bin)	1260 (3x bin)
Initial particle images (no.)	739564	739564	739564	1186871
Final particle images (no.)	14174	15518	27712	77373
Map resolution at FSC=0.143 (Å)	4.54 Å	4.37 Å	4.38 Å	5 Å
**Refinement:**				

Model composition				
Non-hydrogen atoms	46654	46677	94209	113373
Protein residues	5242	5247	10594	12763
Nucleotides	260	260	520	561
B factor (Å^2)				
Protein	318.49	251.41	494.14	281.85
DNA	737.42	404.61	844.53	599.50
R.m.s deviations				
Bond lengths (Å)	0.003	0.003	0.003	0.003
Bond angles (°)	0.648	0.628	0.688	0.624
Validation				
Molprobity score	2.41	2.33	2.45	2.23
Clash score	22.69	20.76	23.91	16.93
Poor rotamers (%)	0.11	0.22	0.34	0.01
Ramachandran plot				
Favoured (%)	89.53	91.15	88.97	91.73
Allowed (%)	10.16	8.66	10.55	8.16
Disallowed (%)	0.31	0.19	0.48	0.11

Supplementary table 3: Nucleosome construct DNA sequences.

DNA length (bp)	DNA sequence
**147**	5’ CCTGGAGAATCCCGGTGCCGAGGCCGCTCAATTGGTCGTAGACAGCTCTAGCACCGCTT AAACGCACGTACGCGCTGTCCCCCGCGTTTTAACCGCCAAGGGGATTACTCCCTAGTCTCC AGGCACGTGTCAGATATATACATCCTG 3’
**153**	5’ ATCCTGGAGAATCCCGGTGCCGAGGCCGCTCAATTGGTCGTAGACAGCTCTAGCACCGCT TAAACGCACGTACGCGCTGTCCCCCGCGTTTTAACCGCCAAGGGGATTACTCCCTAGTCT CCAGGCACGTGTCAGATATATACATCCTGTGAT 3’
**197**	5’ ATCGATGGACCCTATACGCGGCCGCCCTGGAGAATCCCGGTGCCGAGGCCGCTCAATTGGT CGTAGACAGCTCTAGCACCGCTTAAACGCACGTACGCGCTGTCCCCCGCGTTTTAACCGCCAA GGGGATTACTCCCTAGTCTCCAGGCACGTGTCAGATATATACATCCTGTGCATGTATTGAACAGC GACCTGAT 3’

Supplementary table 4: Oligonucleotide sequences.

Oligonucleotides	Sequence (5’ to 3’)
**147-nucleosome-DNA-F**	CCTGGAGAATCCCGGTGCCG
**147-nucleosome-DNA-R**	CAGGATGTATATCAGACACGTGCTGGAGAGGGA
**197-nucleosome-DNA-F**	ATCGATGGACCCTATACG
**197-nucleosome-DNA-R**	ATCAGGTCGCTGTTCAATAC
**Kpn2-AX-Mlu-F**	ccggTCAGGATCTGGTAGCGGTTCCGGATCTCCTAGGTCACCCGGGTCTA
**Kpn2-AX-Mlu-R**	cgcgTAGACCCGGGTGACCTAGGAGATCCGGAACCGCTACCAGATCCTGA
**Ku70-Kpn2-F**	ctctcgTCCGGAGCCACCATGTCAGGGTGGGAGTCATATTACAAAACC
**Ku70-Bcu-R**	ctcgtcACTAGTTCAGTCCTGGAAGTGCTTGGTGAGGGCTTC
**Ku70-R543A-F**	GGGAAAGTTACCAAGgcAAAACACGATAATGAAGGTTCTGGAAGC
**Ku70-R543A-R**	CATTATCGTGTTTTgcCTTGGTAACTTTCCCTTCAGGATTGTAATCTGG
**Ku70-K553A-F**	GGTTCTGGAAGCgcAAGGCCCAAGGTGGAGTATTCAGAAG
**Ku70-K553A-R**	CCACCTTGGGCCTTgcGCTTCCAGAACCTTCATTATCGTG

**Ku70-T577A-F**	GGGTACGCTGGGCAAGTTCgCTGTGCCCATGCTGAAAGAGGCCTGCC
**Ku70-T577A-R**	CATGGGCACAGcGAACTTGCCCAGCGTACCCTTGCTGATG
**Ku70-K596A-F**	CTTACGGGCTGAAGAGTGGgCTGAAGgcGCAGGAGCTGCTGGAAGCCCTC
**Ku70-K596A-R**	GAGGGCTTCCAGCAGCTCCTGCgcCTTCAGcCCACTCTTCAGCCCGTAAG
**Ku70-K596E-F**	CTTACGGGCTGAAGAGTGGgCTGAAGgAGCAGGAGCTGCTGGAAGCCCTC
**Ku70-K596E-R**	GAGGGCTTCCAGCAGCTCCTGCTcCTTCAGcCCACTCTTCAGCCCGTAAG
**HF-Ku70-Kpn2-F**	CCTCGAGGTTTAAACTACGGgatcTCCGGAgccacc
**HF-Ku70-Bcu-R**	CCAGAGGTTGATTATCATATGACTAGTTCAGTCCTGGAAGTGCTTGGTGAGGGCTTC
**HF-GFP-Ku70-LS-F**	cTccggTCAGGATCTGGTAGCGGTTCCGGACCAGATTACAATCCTGAAGGGAAAGTTACC
**HF-GFP-Ku70-P555-S-F**	cTccggTCAGGATCTGGTAGCGGTTCCGGACCCAAGGTGGAGTATTCAGAAGAGGAGC
**HF-Ku70-R554-R**	CCAGAGGTTGATTATCATATGACTAGTTCACCTTTTGCTTCCAGAACCTTCATTATCG
**HF-Ku70-V557-R**	CCAGAGGTTGATTATCATATGACTAGTCTACACCTTGGGCCTTTTGCTTCCAGAACC
